# Single-cell transcriptomics reveals stepwise transformation of epithelial cells into Non-Professional Phagocytes

**DOI:** 10.1371/journal.pgen.1011953

**Published:** 2025-12-04

**Authors:** Caique Almeida Machado Costa, Cristian Alejandro Santiago-Santiago, Yi-Chun Huang, Wu-Min Deng

**Affiliations:** Department of Biochemistry and Molecular Biology, Tulane University School of Medicine, Tulane Cancer Center, United States of America; Academia Sinica, TAIWAN

## Abstract

The differentiation of epithelial cells into Non-Professional Phagocytes (NPPs) is essential for maintaining tissue homeostasis and clearing apoptotic debris. In the *Drosophila* ovary, epithelial follicle cells transform into NPPs following germline cell death, but the genetic mechanisms controlling this transition are not well defined. To investigate these mechanisms, we used a model in which overexpression of the active form of Notch, the Notch Intracellular Domain (NICD), induces a robust epithelial-to-NPP transition. Using single-cell RNA sequencing and trajectory analysis, we identified three transcriptional phases of NPP maturation: an early stage of metabolic activation, an intermediate stage enriched in genes related to migration and cytoskeletal remodeling, and a late stage marked by autophagy-related gene expression. These transcriptomic patterns were validated by immunostaining. SCENIC and ChiP-seq analyses identified the JNK effector Jun-related antigen (Jra) and its predicted targets, *Arp2* and *Arp3*, which encode components of the Arp2/3 complex, as regulators of cytoskeletal remodeling. Functional assays confirmed that the JNK–Jra–Arp2/3 axis is required for cytoplasmic expansion and debris clearance during NPP differentiation.

## Author summary

Our immune system relies on specialized cells called phagocytes to capture and remove harmful bacteria, viruses, and dying cells. However, in some situations, other cell types that are not normally phagocytic, such as epithelial cells, can temporarily take on this cleaning role. These cells, known as Non-Professional Phagocytes, help reduce the burden on immune cells and are thought to play important roles in preventing diseases like cancer, neurodegeneration, and autoimmunity. Yet, it has remained unclear how ordinary epithelial cells acquire this ability. In our study, we used the fruit fly *Drosophila* to create a model where we could trigger this transformation by activating a signaling pathway called Notch. Using single-cell sequencing, we identified the specific genes and molecular changes that occur as epithelial cells gain phagocytic properties. We discovered that two key regulators, Jra, and the Arp2/3 complex, work together to reshape the cell’s structure and enable this transformation. Our findings provide insight into how tissues adapt to maintain balance and remove unwanted cells.

## Introduction

Phagocytosis of dead cell debris is a fundamental process to keep tissue homeostasis and prevent autoimmune disorders [3], neurodegenerative diseases [4] and cancer [5]. While professional phagocytes, including macrophages and neutrophils are specialized on this function, a group of cells known as NPPs, including epithelial cells from lung, intestine, retinal pigment and fibroblasts, display a fundamental role on debris clearance from apoptotic cells [6,7].

*Drosophila* ovaries represent an ideal model system for studying the transition of epithelial cells into NPPs. Each ovary is composed of multiple egg chambers, which progress through 14 distinct developmental stages. These are typically classified into early (stages 1–6), mid (stages 7–9), and late stages (stages 10–14), ultimately culminating in a mature egg ready for oviposition. Each egg chamber contains germline cells surrounded by an epithelial monolayer of follicle cells [8]. When facing environmental stressors such as starvation [9,10,11], cold acclimation [12,13], or radiation [14], mid-stage egg chambers trigger germline cell death as a strategy to prevent defective egg development [15]. Under these conditions, follicle cells switch their epithelial role to become NPPs, phagocytizing apoptotic germline debris, completing a process previously defined in our study as Germline Death And Clearance (GDAC) [16]. This transition is promoted by key molecular events that involve the activation of JNK signaling [9,11], upregulation of phagocytic receptors, modulation of GTPases, and cytoskeleton remodeling, which collectively drive the morphological changes associated with NPP differentiation. These changes include progressive NPPs’ cytoplasmic expansion towards the germline area, uptake of apoptotic debris and ultimately complete occupation of the germline-cell area by NPPs [10].

Our recent study demonstrated that activation of the Notch signaling pathway is both necessary and sufficient to drive the differentiation of follicle cells into NPPs, in part by promoting polyploidization and activating the JNK pathway [16]. Building upon our previous findings, we employed a constitutively active *NICD* overexpression (OE) system that promotes GDAC [16] to investigate the transcriptional programs underlying epithelial-to-NPP differentiation in mid-stage egg chambers. This system not only recapitulates key features of starvation-induced NPP differentiation but also induces GDAC at a higher frequency, providing a powerful framework for dissecting the transcriptomic changes that govern follicle cell differentiation into NPPs.

To further elucidate the transcriptional dynamics underlying epithelial-to-NPP transitions, we employed single-cell RNA sequencing (scRNA-seq) to track gene expression changes at single-cell resolution. Unlike bulk RNA sequencing, which averages gene expression across heterogeneous cell populations, scRNA-seq enables the transcriptomic characterization at a single cellular resolution. By applying our *NICD*-OE model, we compared the transcriptional profiles of *NICD-*OE with well-fed wild-type follicle cells, which allowed us to define distinct clusters belonging to differentiated NPPs. These clusters were further used to capture distinct differentiation steps taken by follicle cells transitioning to NPPs and their respective transcriptional changes required for this transition. In addition, our analysis identified key transcriptional factors such as Jra, allowing us to predict its downstream target genes, including components of the Arp2/3 complex, whose function in cytoskeletal rearrangements during NPP differentiation was subsequently validated.

## Results

### scRNA-seq transcriptomics reveals *NICD* OE-derived NPPs

**Fig 1:**
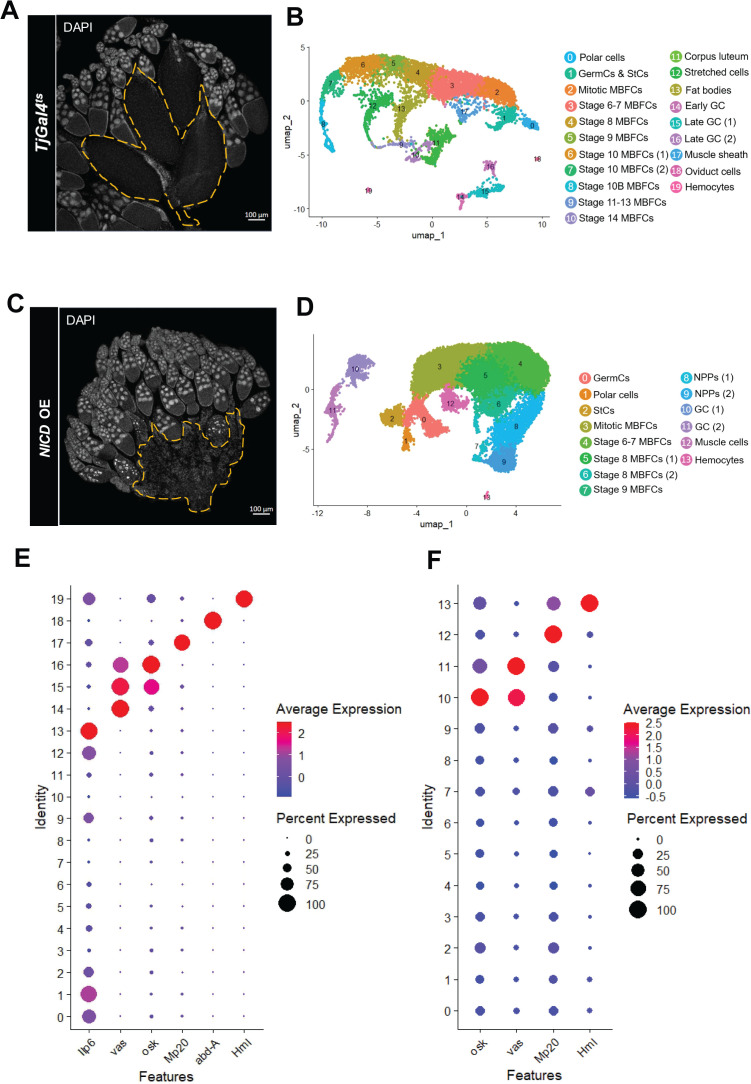
Single-cell RNA-seq analysis of adult *Drosophila* ovaries in *wild-type* and *NICD-*OE backgrounds. (A, C) Confocal images of adult *Drosophila* ovaries expressing the *Tj-GAL4*^*ts*^ driver, which is phenotypically equivalent to the *wild-type* (*w^1118^*) and serves as the control condition for panel (C). In (A), the yellow dashed line outlines the region occupied by fully differentiated eggs marking oogenesis completion in a *wild-type* ovary. In contrast, (C) shows a *Tj-GAL4*^*ts*^*>UAS-NICD* ovary, where the yellow dashed line highlights the absence of late-stage egg chambers due to oogenesis arrest at stage 9, a phenotype induced by *NICD* OE [16]. DAPI staining displays cell nuclei. (B, D) UMAP projections showing the distribution of ovarian cell populations in (B) *wild-type* flies and (D) *NICD*-OE flies, highlighting shifts in population distribution between the two datasets. Abbreviations: GermCs, Germarium Cells; StCs, Stalk Cells; MBFCs, Main Body Follicle Cells. (E, F) Dot plots illustrating the expression of cell type-specific markers used to assign non-epithelial-cell related clusters in both *w^1118^* and *NICD*-OE datasets.

To investigate the transcriptomic changes underlying the differentiation-promoting effect of *NICD*-OE in follicle cells into NPPs at single-cell resolution, we analyzed scRNA-seq data from *wild-type* (*w^1118^*) and *NICD*-OE ovaries (Fig 1A, C). Using Seurat [17], we clustered the cells (Fig 1B, D) and assigned identities based on established cell type-specific markers for *Drosophila* ovarian tissue [18,2,1,19] (S1A-C Fig).

In the wild-type dataset, 20 clusters were identified, representing cells from the 14 developmental stages of egg chambers as well as adjacent tissues, including oviduct cells, muscle sheath, hemocytes, and adipocytes (Fig 1B). In the *NICD*-OE dataset, 14 clusters were identified, corresponding to cells from egg chambers up to early stage 10 (Fig 1D). Cells expressing the late-stage marker *Femcoat* were largely absent (S1B Fig), consistent with the oogenesis arrest at stage 9 caused by *NICD*-OE-driven GDAC [16].

Within the *NICD*-OE dataset, clusters 8 and 9 attracted particular attention due to their proximity to follicle cells clusters belonging to stage 8 and 9 egg chambers (clusters 5–7) but lacked stage-specific markers such as *Vitelline membrane-like* (*Vml*), *palisade* (*psd*), and *defective chorion 1* (*dec-1*), suggesting a distinct cellular identity (S1B Fig).

To better characterize the unique effect promoted by *NICD* OE to promote the differentiation of epithelial follicle cells into NPPs, we decided to identify and remove non-epithelial cell types originating from adjacent tissues in both datasets by analyzing the expression of cell-type markers, such as *Insulin-like peptide 6* (*Ilp6*), which is associated with adipocytes and highly expressed on *wild-type* dataset clusters 13, (2.990313449 avg_avg_log2FC, padj = 1.34E-218), *vasa* (*vas*), cluster 14 (4.829041 avg_log2FC, padj = 1.32E-276), 15 (4.17325 avg_log2FC, padj = 0) and 16 (2.295376 avg_log2FC, padj = 1.47E-37), *oskar* (*osk*), cluster 15 (2.067233 avg_log2FC, padj = 1.25E-96), 16 (6.976927 avg_log2FC, padj = 1.59E-124), *Muscle protein 20* (*Mp20*), cluster 17 (5.128807 avg_log2FC, padj = 1.76E-151), *abdominal A* (*abd-A*), cluster 18 (9.525009 avg_log2FC, padj = 0), *Hemolectin* (*Hml*), cluster 19 (10.63374 avg_log2FC, padj = 0) (Fig 1E, S1 Table). On *NICD*-OE dataset, *osk*, cluster 10, (6.149274165 avg_log2FC, padj = 0), *vas*, cluster 10, (3.408142877 avg_log2FC, padj = 0), cluster 11, (4.026276583 avg_log2FC, padj = 0), *Mp20*, cluster 12, (4.985966844 avg_log2FC, padj = 0), *Hml*, cluster 13, (12.27544819 avg_log2FC, padj = 0) (Fig 1F, S2 Table).

**Fig 2:**
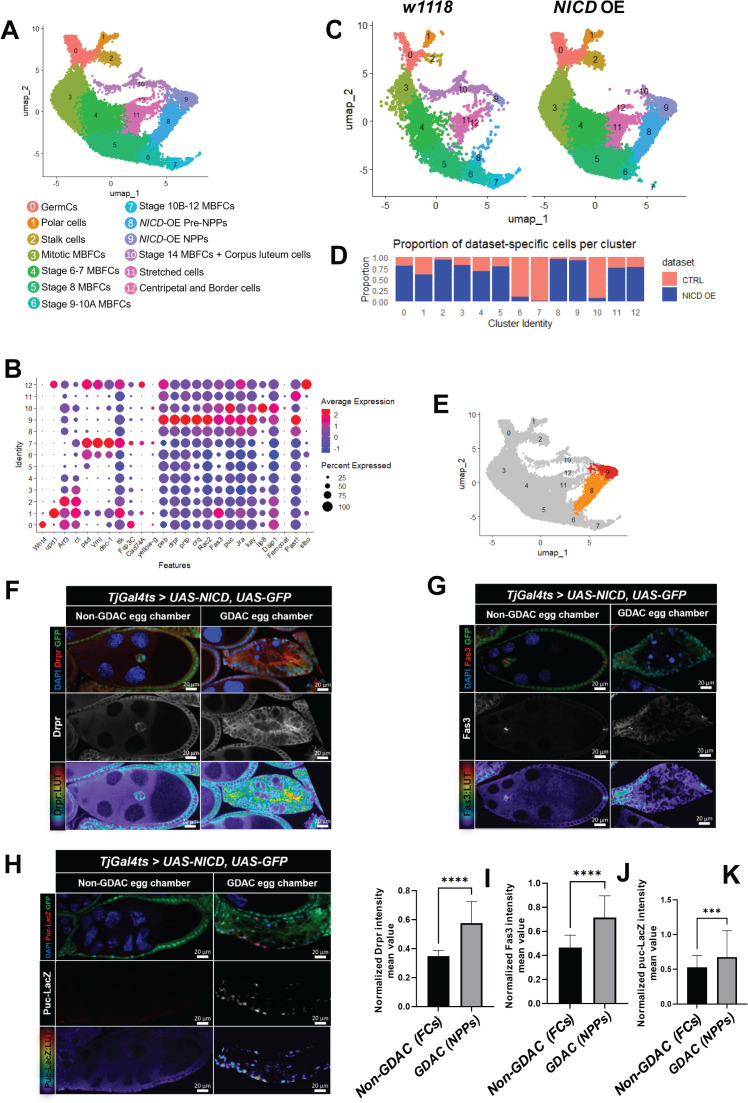
Integration of *w^1^^118^* and *TjGal4*^*ts*^*>UAS-NICD* datasets reveals unique NPP clusters derived from *NICD* overexpressing cells. (A) UMAP projection of integrated follicle cells from *w^1118^* and *TjGal4*^*ts*^*>UAS-NICD* datasets, illustrating the overall cellular landscape. (B) Dot plot displaying the expression of cell type-specific markers across integrated data set cluster numbers. (C) UMAP embedding showing cluster distribution by sample ID, with *w^1118^* cells from the *wild-type* dataset and *NICD-*OE cells from the *TjGal4*^*ts*^*>UAS-NICD* dataset (D) Bar graph representing the relative percentage of cells in each cluster, with bars segmented by dataset of origin: *w^1118^* (salmon) and *NICD* OE (navy blue). (E) UMAP projection illustrating the overlap between *w^1118^* and *NICD*-OE cells, with clusters highlighted in orange and red, predominantly derived from the *NICD-*OE dataset. (F-H) Confocal images showing staining of specific NPP markers, including Drpr, puc-lacZ and Fas3 in *NICD-*OE egg chambers undergoing GDAC compared to those not undergoing GDAC, named as GDAC egg chambers and Non-GDAC egg chambers respectively. DAPI stains for cells nuclei and GFP marks *TjGal4*^*ts*^ transcriptional activation. Rainbow2 LUT (Lookup Table) visualizes intensity variations in images by mapping different pixel values to a color scale, enhancing the comparison of staining intensity between images. (I-K) Bar plots with interquartile range showing the median for the normalized intensity mean values of Drpr (I), Fas3 (J), and puc-LacZ (K) in GDAC versus Non-GDAC egg chambers. Sample sizes for GDAC are N = 75 (I), N = 58 (J), and N = 73 (K), while Non-GDAC sample sizes are N = 52 (I), N = 51 (J), and N = 52 (K). P-values obtained from Mann Whitney test are indicated by **** (p ≤ 0.0001 for I and J) and *** (p ≤ 0.001 for K) above each plot.

Following the removal of non-epithelial cell types, we integrated the *NICD*-OE and *wild-type* datasets to highlight the transcriptomic changes caused by *NICD*-OE. This integrated dataset revealed 13 clusters (Fig 2A), which were identified using established cell type-specific markers [18,2,1,19] (Fig 2B). To delineate the transcriptional alterations driven by *NICD*-OE, we separated the integrated clusters into cells originating specifically from the *w^1118^* (control) and *NICD*-OE (stimulated) datasets (Fig 2C). We then analyzed the proportion of cells from each dataset within each cluster.

Consistent with the known biological effects of *NICD* OE on stopping oogenesis at stage 9 egg chambers, clusters 6, 7, and 10, which correspond to cells from egg chambers at stages 9–14 and corpus luteum cells, were predominantly represented in the *w^1118^* dataset. Interestingly, clusters 8 and 9 were predominantly unique to the *NICD*-OE dataset (Fig 2D, E) and displayed distinct genetic signatures that differed from other known oogenesis cell types (Fig 2B, S3 Table).

To identify the cell types represented by clusters 8 and 9, we performed differential gene expression analysis, comparing their genetic profiles to the remaining 11 clusters. This analysis revealed significant upregulation of genes related to NPP phagocytosis, including the phagocytic receptors *croquemort* (*crq*) [20] (3.571624 avg_log2FC, padj = 0) and *draper* (*drpr*) [9,15,10,11] (1.417401 avg_log2FC, padj = 3.74E-180), Drpr’s ligand, *pretaporter* (*prtp*) [21], (0.98939 avg_log2FC, padj = 5.47E-58), the GTPase *Rac2* [10,15] (1.729876 avg_log2FC, padj = 0), the JNK-regulated gene *puckered* (*puc*) (0.847344 avg_log2FC, padj = 1.29E-91) [9,15,10,11] and downstream transcription factors *Jun-related antigen* (*Jra*) (0.929437 avg_log2FC, padj = 2.14E-87) and *kayak* (*kay*) (1.266146 avg_log2FC, padj = 4.99E-163) coding genes [22]. Additionally, *Fasciclin 3* (*Fas3*), a gene previously unreported in this context, was distinctly expressed and enriched in NPPs (1.7174 avg_log2FC, padj = 0) (Fig 2B, S3 Table).

To validate these findings, we performed immunofluorescence analysis to examine the expression and fluorescence intensity patterns of Drpr, Fas3, and Puc in *NICD-*OE-derived NPPs compared to control follicle cells (FCs) from egg chambers not undergoing GDAC. Quantification of normalized fluorescence intensity revealed significantly higher expression of Drpr (median FC = 0.35 in FCs vs. 0.58 in NPPs, n = 52 and 76 respectively, p < 0.0001) (Fig 2F, I), Fas3 (0.47 in FCs vs. 0.71 in NPPs, n = 51 and 58, p < 0.0001) (Fig 2G, J), and puc (0.53 in FCs vs. 0.67 in NPPs, n = 52 and 73, p = 0.0005) (Fig 2H, K). These results confirm the distinct and enriched expression of Drpr, Fas3, and Puc in *NICD-*OE-derived NPPs. Together, these findings support the conclusion that clusters 8 and 9 represent a population of *NICD*-OE-induced NPPs with a unique transcriptomic identity that sets them apart from other follicle cell types.

### Distinct *NICD*-OE clusters exhibit transcriptomic changes associated with successive stages of NPP differentiation

**Fig 3:**
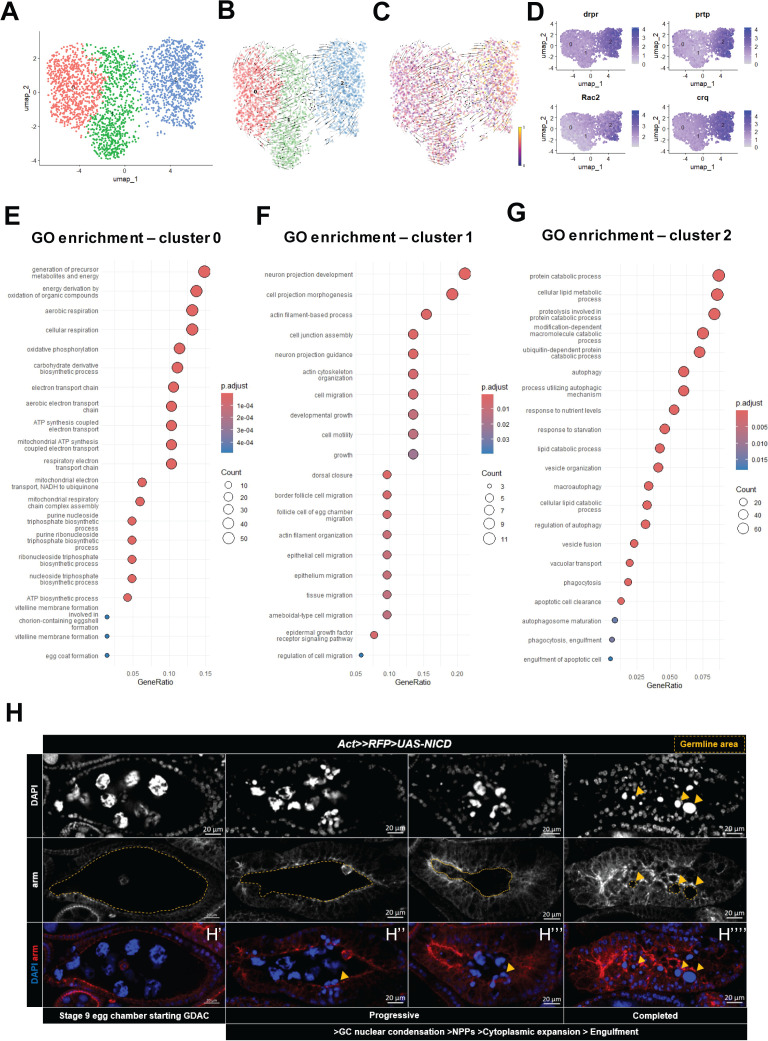
*NICD*-OE-derived NPP clusters exhibit distinct gene expression profiles and differentiation trajectories. (A) UMAP projection of re-embedded *Tj-Gal4*^*ts*^*>UAS-NICD* derived NPP clusters, highlighting their unique cellular organization. (B) RNA velocity vectors overlaid on the UMAP plot, illustrating inferred cellular lineages and differentiation trajectories. (C) Pseudotime analysis showing the progressive differentiation of *Tj-Gal4*^*ts*^*>UAS-NICD*-derived NPPs clusters. (D) Differentially expressed gene markers identified in the re-embedded NPP clusters, highlighting key genes associated with NPP differentiation. (E-G) Differential gene expression analysis for clusters 0, 1 and 2, reflecting distinct transcriptomic profiles at successive stages of NPP differentiation. Dot plots summarizing GO enrichment analysis for cluster 0, 1 and 2, respectively. The x-axis represents the gene ratio, while the y-axis lists enriched GO Biological Process terms. Dot size corresponds to the number of genes associated with each term, and the color gradient represents the adjusted p-value (*p.adjust*), with darker shades indicating higher statistical significance. (H) Confocal images depict distinct morphological changes that characterize the differentiation stages of *NICD*-OE NPPs during GDAC. These stages align with the biological processes assigned to the differentially expressed genes predominantly expressed in each NPP cluster. Yellow arrows indicate the nuclei of dying germline cells engulfed by NPPs. Yellow dashed line displays germline area during different stages of NPP differentiation. Nuclei are stained with DAPI (blue), and the cell membrane is marked with Armadillo (red).

During germline cell death, NPPs undergo a series of gradual morphological changes, including cytoplasmic expansion and cytoskeleton remodeling that enable the digestion of germline cell debris [9,15,10,11,23]. To determine whether our analysis could capture this chronological differentiation process in *NICD*-OE-derived NPPs clusters, we focused on clusters 8 and 9, due to the predominance of cells derived from the *NICD*-OE data set. We re-clustered these cells and identified three distinct sub-clusters labeled 0, 1, and 2 (Fig 3A).

To test whether these sub-clusters reflected successive stages of NPP differentiation, we performed RNA velocity and pseudotime analyses. The RNA velocity vectors demonstrated a clear directionality, starting from cluster 0, progressing to cluster 1, and culminating in cluster 2 (Fig 3B). Pseudotime analysis further confirmed this alignment, with cluster 2 displaying the highest pseudotime values (represented in yellow), indicative of the most advanced differentiation stage (Fig 3C).

Consistent with this, cluster 2 displayed the highest expression levels for NPP markers, including the phagocytic receptors *crq* (2.62 avg_avg_log2FC, padj = 2.12E-257) [20] and *drpr* (1.63 avg_avg_log2FC, padj = 3.41E-72) [9,21,11], Drpr’s ligand, *prtp*, (2.22 avg_avg_log2FC, padj = 1.70E-117) [21], and the GTPase *Rac2* (1.22 avg_avg_log2FC, padj = 1.17E-57) [24], supporting cluster 2, as the NPPs on their most advanced differentiation stage (Fig 3D, S4 Table).

To further investigate whether the transcriptional profiles of these subclusters align with the known morphological and functional stages of NPP maturation, we performed differential gene expression analysis followed by Gene Ontology (GO) enrichment analysis [25]. Cluster 0 was enriched for genes associated with egg coat formation (GO:0035803; p.adj = 0.00049), vitelline membrane formation (GO:0030704; p.adj = 0.00049) and vitelline membrane formation involved in chorion-containing eggshell formation (GO:0007305; p.adj = 0.00049) (Fig 3E), suggesting that these cells retain characteristics of mid-stage follicle cells and represent an early transitional state toward NPP identity.

Moreover, cluster 0 cells displayed enriched expression of genes related to mitochondrial energy metabolism: aerobic respiration (GO:0009060; p.adj = 5.61E-33), mitochondrial ATP synthesis coupled electron transport (GO:0042775; p.adj = 8.98E-29), mitochondrial electron transport, NADH to ubiquinone (GO:0006120; p.adj = 1.52E-19), mitochondrial respiratory chain complex assembly (GO:0033108; p.adj = 1.20E-14) (Fig 3E, S5 Table). We validated the increase in mitochondrial activity and energy demand in both starvation and *NICD*-OE NPPs models, by immunostaining with the ATP5A antibody, which targets the alpha subunit of ATP synthase (complex V), a key component of the mitochondrial oxidative phosphorylation machinery [26]. Follicle cells undergoing the transition to NPPs exhibited significantly elevated ATP5A fluorescence intensity compared to control follicle cells in both *NICD*-OE (S2A, S2B Fig; mean FCs = 0.37 vs. 0.71 in NPPs, n = 91 and 73, p < 0.0001) and starvation model (S2C, S2D Fig; median FCs = 0.39 vs. 0.85 in NPPs, n = 75 and 80, p < 0.0001). Taken together, the transcriptional expression profile of cluster 0 cells resembles epithelial follicle cells undergoing normal development that have begun metabolic reprogramming through enhanced mitochondrial activity, likely to meet the increased energy demands required for their emerging phagocytic function as NPPs (Fig 3H’, S3A’ Fig).

Analysis of cluster 1 revealed upregulation of genes involved in cell projection morphogenesis (GO:0048858; p.adj = 0.00079), actin cytoskeleton organization (GO:0030036; p.adj = 0.00494), cell migration (GO:0016477; p.adj = 0.00580) and growth (GO:0040007; p.adj = 0.0214) (Fig 3F, S6 Table). These biological processes are consistent with the behavior of early-stage NPPs, which exhibit cytoplasmic expansion and actively invade the germline area to engulf apoptotic debris following germline cell death [10] (Fig 3H’’, H’’’, S3A’’ Fig).

Finally, cluster 2 exhibited transcriptional enrichment in pathways related to protein degradation and nutrient stress responses, including protein catabolic process (GO:0030163, padj = 6.22E-13), autophagy (GO:0006914, padj = 5.25E-13), cellular response to starvation (GO:0009267, padj = 3.48E-06), regulation of autophagy (GO:0010506, padj = 1.00E-06), and apoptotic cell clearance (GO:0043277, padj = 1.85E-06) (Fig 3G, S7 Table). These processes reflect the metabolic shift required by differentiated NPPs to adapt to nutrient deprivation and perform the digestion of cellular debris, consistent with their fully differentiated state [27,28,29,30] (Fig 3H”, S3A”’ Fig).

### Autophagy is required for *NICD*-OE-derived NPPs to perform germline cell debris clearance

The enrichment of autophagy-related genes in cluster 2 (Fig 3G), representing the most advanced stage of NPP differentiation, aligns with previous studies that report the activation of autophagy in mid-stage egg chambers under amino acid deprivation [27,28,29,30]. These studies state autophagy as essential for the proper clearance of germline cell debris by NPPs.

**Fig 4:**
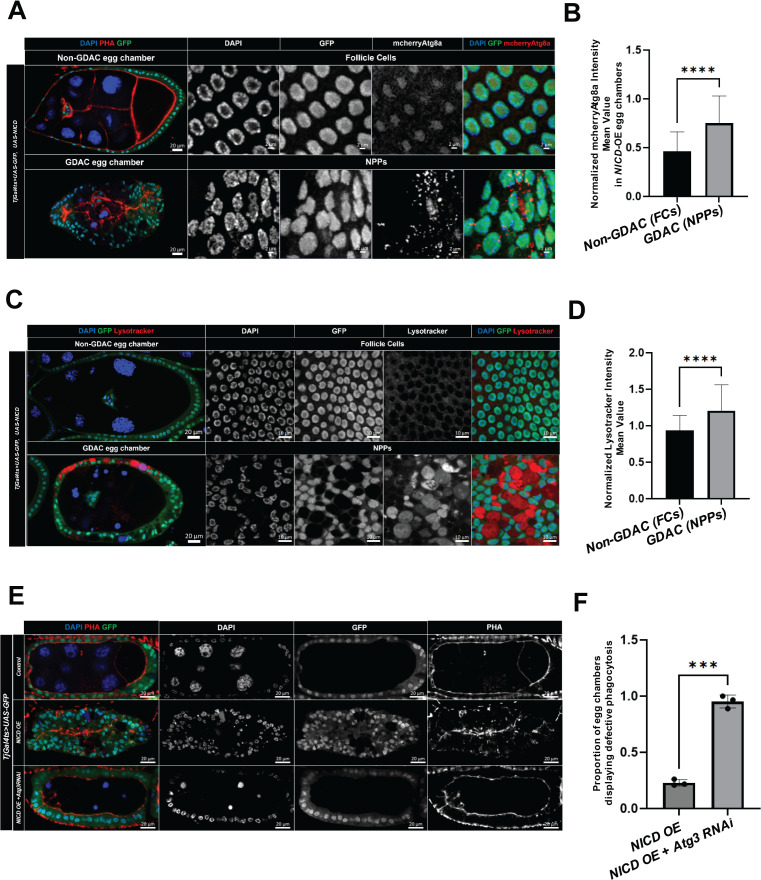
Autophagy is enriched in NPPs and required for effective germline debris clearance. (A) Confocal images of *Tj-Gal4^ts^>UAS-GFP, UAS-NICD* egg chambers undergoing GDAC and Non-GDAC conditions, showing expression levels of the autophagy reporters *mCherry-Atg8a* in follicle cells versus NPPs. (B) Bar plots with error bars showing normalized intensity median values for *mCherry-Atg8a* in GDAC versus Non-GDAC egg chambers under *NICD*-OE condition. Sample sizes are N = 114 and N = 63 for GDAC and Non-GDAC, respectively. P-values obtained from Mann Whitney test are indicated by **** (p < 0.0001) above the plot. (C) Confocal images of *Tj-Gal4ts, UAS-GFP > UAS-NICD* under Non-GDAC and GDAC conditions. LysoTracker staining highlights autophagy-related activity in follicle cells and NPPs. (D) Bar plot with error bars showing normalized Lysotracker intensity mean values in GDAC versus Non-GDAC egg chambers under *NICD*-OE condition. Sample sizes: *NICD*-OE GDAC (N = 49), Non-GDAC (N = 79). P-value from Mann-Whitney tests is indicated as **** (p < 0.0001). (E) Confocal images comparing *Tj-Gal4^ts^>UAS-GFP, UAS-NICD* versus *Tj-Gal4^ts^>UAS-GFP, UAS-NICD + UAS-Atg3RNAi* egg chambers. DAPI stains cell nuclei, GFP marks *Tj-Gal4^ts^* transcriptional activation and Phalloidin (PHA), actin filaments. (F) Bar plots with error bars showing the percentage of egg chambers displaying defective phagocytosis for each genotype: *NICD*-OE (N = 52), *NICD*-OE* + Atg3RNAi* (N = 58). A p-value obtained from t-test is indicated by *** (p ≤ 0.001).

To determine whether autophagy plays a similar role in *NICD*-OE-derived NPPs, we first confirmed the enrichment of the autophagy reporter *mCherry-Atg8a* in these cells. *NICD*-OE-induced NPPs exhibited significantly higher *mCherry-Atg8a* signal compared to control follicle cells (Fig 4A-B; median FCs = 0.40 vs. 0.71 in NPPs, n = 63 and 114, respectively, p < 0.0001). This result was corroborated using Lysotracker staining, another autophagy-related marker [31], suggesting increased autophagic activity in *NICD*-OE-derived NPPs. (Fig 4C, D; median FCs = 0.90 vs. 1.16 in NPPs, n = 79 and 49, p < 0.0001).

To test whether autophagy is functionally required for germline debris clearance in *NICD*-OE NPPs, we depleted *Autophagy-related gene 3* (*Atg3*), which encodes a key protein required for autophagosome formation. Depletion of *Atg3* led to defective phagocytosis in *NICD*-OE-derived NPPs. This defect was characterized by the accumulation of unprocessed, condensed germline DNA debris in the germline area. Furthermore, these cells failed to expand their cytoplasm and engulf the germline cell area, critical steps required for the complete phagocytosis of germline cell debris [15,10] (Fig 4E-F; mean *NICD*-OE = 0.229, Replicate 1: 0.201, N = 43, Replicate 2: 0.218, N = 55, Replicate 3: 0.262, N = 61; mean *NICD* OE* + Atg3 RNAi = *0.951, Replicate 1: 1, N = 47, Replicate 2: 0.888, N = 72, Replicate 3: 0.966, N = 60, p ≤ 0.001).

Together, these findings reveal a transcriptional enrichment of autophagy-related genes in differentiated NPPs, supporting our proposed model (S3 Fig) and indicating that autophagy is essential for germline cell debris clearance in *NICD*-OE–derived NPPs. This is consistent with previous reports in starvation-induced NPPs [27,28,29,30]. The resemblance between these models further underscores their similarity and reinforces the utility of *NICD* OE for identifying shared mechanisms between the two systems.

### SCENIC analysis identifies the JNK pathway transcription factor Jra as a regulator of NPP differentiation

**Fig 5:**
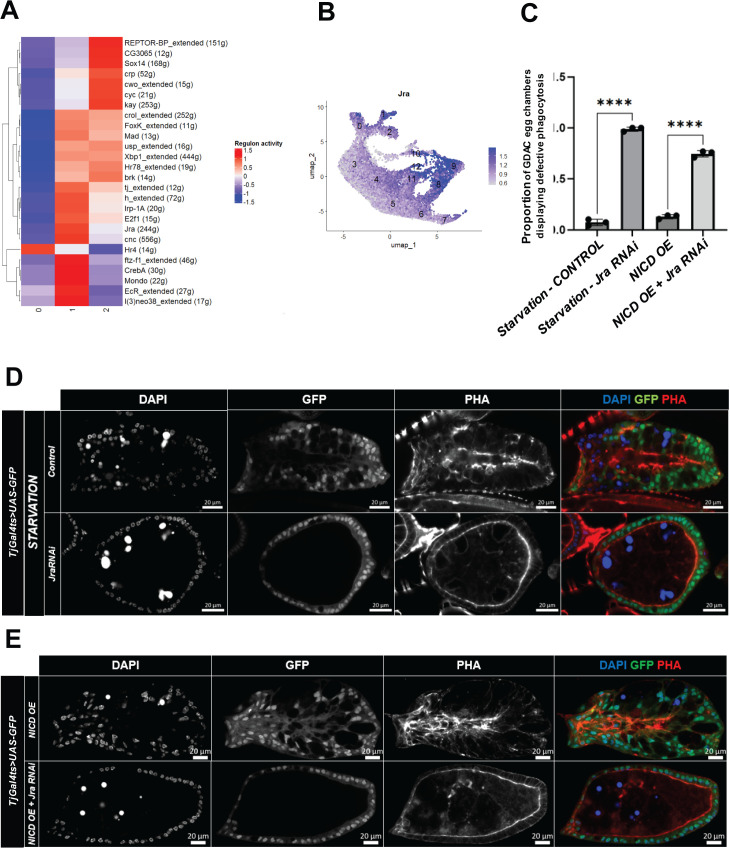
SCENIC analysis reveals key transcription factors driving NPP differentiation. (A) Heatmap of the scaled and centered activity scores of regulons in clusters 0, 1, and 2. Both clusters (columns) and regulons (rows) are hierarchically clustered, revealing the relative similarity in regulon activity across these clusters. (B) Feature Plot illustrating the gene expression of *Jra* across cell clusters in the integrated dataset. Cluster numbers correspond to the identities shown in Fig 2A (C) Bar plots with error bars showing the percentage of egg chambers exhibiting defective phagocytosis in each replicate (Rep.) across different genotypes: mean *Jra RNAi -* Starvation = 0.9881 (Replicate 1: 0.9643, N = 28, Replicate 2: 1, N = 68, Replicate 3: 1, N = 72), mean Control - Starvation = 0.073 (Replicate 1: 0.042, N = 24, Replicate 2: 0.1034, N = 29, Replicate 3: 0.0741, N = 27), mean *NICD*-OE + *JraRNAi* = 0.7470 (Replicate 1: 0.7699, N = 452, Replicate 2: 0.76, N = 550, Replicate 3: 0.7111, N = 623), mean *NICD*-OE = 0.1295 (Replicate 1: 0.1414, N = 99, Replicate 2: 0.1436, N = 57, Replicate 3: 0.1068, N = 103). P-values obtained from t-tests are indicated by **** (p ≤ 0.0001). (D, E) Confocal images illustrating GDAC induction by starvation stimuli (D) or *NICD*-OE (E), comparing the effects of *Jra* depletion (RNAi) under both starvation conditions (D) and *NICD*-OE (E). DAPI stains for cells nuclei, GFP reports *TjGal4*^*ts*^ driver activity and PHA displays actin filaments.

After identifying *NICD*-OE-derived NPPs and their respective differentiation stages, we leveraged the advanced analytical capabilities of scRNA-seq, particularly Single-Cell rEgulatory Network Inference and Clustering (SCENIC), to uncover transcription factors potentially regulating NPP differentiation across different stages. Our analysis identified 11 regulons with distinct activity patterns across NPPs clusters 0, 1, and 2 (Fig 5A, S8 Table). Among these, we focused on Jra, the downstream transcription factor of the JNK signaling pathway, which has been established as essential for NPP differentiation [9,11]. However, the precise role of JNK in regulating the differentiation of follicle cells into NPPs remains poorly understood.

To explore Jra’s role, we first validated the enriched expression of *Jra* observed in SCENIC analysis (Fig 5A) and NPPs clusters (Fig 5B) by examining Jra:GFP expression in *NICD-OE* (S4A, S4B Fig; early-stage egg chambers, median = 0.53, N = 71; mid-stage egg chambers, median = 0.74, N = 71; and GDAC egg chambers, median = 1.35, N = 71) and starvation-induced NPPs (S4C, S4D Fig; early-stage egg chambers, median = 0.72, N = 44; mid-stage egg chambers, median = 0.92, N = 45; and GDAC egg chambers, median = 1.5, N = 22). As expected, in both *NICD*-OE and starvation models, Jra:GFP intensity was significantly higher in NPPs compared to control follicle cells from early- and mid-stage egg chambers, confirming the expression pattern suggested by scRNA-seq analysis (Fig 5B).

Next, we tested the effect of depleting *Jra* on NPPs using RNAi, which effectively suppressed JNK pathway activity, as indicated by reduced TRE:GFP expression (S5A, S5B Fig; median *Jra RNAi* = 0.66 vs. 0.95 in *Control*, N = 35 and 34 respectively, **** (p ≤ 0.0001)). *Jra*-depleted cells failed to invade germline area and expand their cytoplasm to engulf apoptotic debris, leaving uncleaned debris in both starvation and *NICD*-OE model systems (Fig 5C, D, E; mean *Control-Starvation *= 0.0731, Replicate 1: 0.042, N = 24, Replicate 2: 0.1034, N = 29, Replicate 3: 0.0741, N = 27; mean *Jra RNAi* = 0.9881, Replicate 1: 0.9643, N = 28, Replicate 2: 1, N = 68, Replicate 3: 1, N = 72; mean *NICD* OE = 0.1295, Replicate 1: 0.1414, N = 99, Replicate 2: 0.1436, N = 57, Replicate 3: 0.1068, N = 103; mean *NICD* OE* + Jra RNAi *= 0.7470, Replicate 1: 0.7699, N = 452, Replicate 2: 0.76, N = 550, Replicate 3: 0.7111, N = 623, **** (p ≤ 0.0001)). In agreement with previous studies showing that JNK depletion disrupts NPP differentiation [9,11].

**Fig 6:**
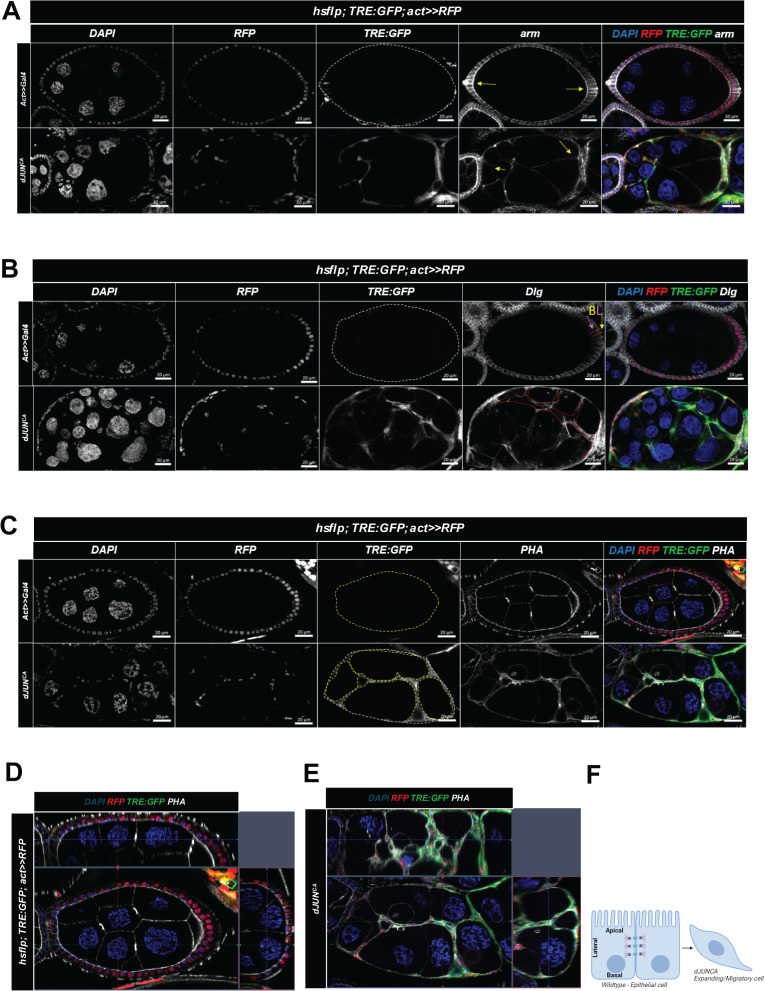
JNK activation induces follicle cell invasion and cytoplasmic expansion. (A-C) Confocal images comparing *dJUN*^*CA*^-expressing egg chambers to control egg chambers (*hsflp; TRE:GFP; act>>RFP*), with staining for Armadillo (A), Dlg (B), and PHA (C). *TRE:GFP*-positive cells indicate JNK signaling activation, DAPI marks cell nuclei, and RFP identifies cells with an active *act>>Gal4* driver. Armadillo highlights cell junctions, Dlg indicates basolateral cell polarity, and PHA visualizes actin filaments. (A) Yellow arrows highlight differences in cell membrane organization as revealed by armadillo staining. (B) Yellow and pink arrows indicate basolateral (BL) polarity in control epithelial cells, while the red dashed line marks disrupted polarity in *dJUN*^*CA*^-expressing cells. (C) Yellow dashed lines delineate the germline cell area in control versus *dJUN*^*CA*^-expressing egg chambers, illustrating germline area invasion by *dJUN*^*CA*^-expressing follicle cells. (D, E) Orthogonal projections of confocal images showing differences in follicle cell morphology and structural organization between control (D) and *dJUN*^*CA*^-expressing egg chambers (E). (F) Schematic illustration depicting the stretched, migratory morphology of *dJUN*^*CA*^-expressing cells compared to *wild-type* epithelial cells.

In contrast, overexpression of a constitutively active form of *Jra* (*dJun*^*CA*^) resulted in cells exhibiting a stretched morphology, with enhanced cytoplasmic expansion and invasion into germline cells (Figs 6A-F; S5C; mean *dJunCA* = 0.9485, Replicate 1: 0.9434, *N* = 53; Replicate 2: 0.9535, *N* = 43; Replicate 3: 0.9487, *N* = 39) in a similar manner to the effects previously observed with *hemipterous* (*hep*^*CA*^) activation [9]. However, no invasive phenotype was observed in control ovarioles (Fig 6A-F; S5C Fig; *N* = 62, 37, and 55, respectively, ****p ≤ 0.0001).

Interestingly, immunostaining analysis using antibodies against Armadillo (Arm, cell membranes) (Fig 6A), Discs large 2 (Dlg, basolateral polarity) (Fig 6B), and phalloidin (PHA, actin filaments) (Fig 6C-E), revealed that Jra-overexpressing cells displayed enhanced rearrangements of these features compared to control follicle cells. These changes resemble key characteristics of epithelial cells transitioning to a mesenchymal migratory state [32,18,33], further reinforcing the role of JNK in regulating cytoskeleton remodeling.

### Jra regulates cytoskeleton rearrangement during NPP differentiation

To gain insights into how Jra orchestrates NPP differentiation, we analyzed its regulons, focusing on predicted downstream-regulated genes and their functions. Gene Ontology (GO) analysis revealed that Jra predicted targets, enriched in NPP cluster 1 (AUC: cluster 0 = 0.0158, cluster 1 = 0.0207, cluster 2 = 0.0159; Z-scored activity: cluster 0 = – 0.60, cluster 1 = 1.15, cluster 2 = –0.56) are involved in processes such as actin cytoskeleton organization (GO:0030036; p.adj = 1.22E-06), actin filament-based process (GO:0030029; p.adj = 1.66E-07), and actin filament organization (GO:0007015; p.adj = 0.00016) (S6A, S6B Fig, S9 Table). This aligns with our previous differential gene expression analysis pointing NPP cluster 1 as being associated with genes related to cell growth, migration, and actin cytoskeleton remodeling (Fig 3F).

These processes are essential for NPPs as they expand their cytoplasm and invade the germline cell area to engulf and clear apoptotic debris. Consistently, *Jra*-depleted NPPs exhibited impaired cytoplasmic expansion, reduced invasion capacity, and an inability to fully clear germline cell debris (Fig 5C, D, E). Conversely, *Jra*-overexpressing follicle cells invaded the germline region, exhibited actin-cytoskeleton rearrangements and displayed a stretched morphology (Fig 6A-F; S5C Fig). These findings suggest that Jra’s downstream targets are involved in cytoskeletal rearrangement of follicle cell maturating into NPPs.

### Functional analysis identifies the Arp2/3 complex as a key regulator of cytoskeleton remodeling in NPPs

To investigate the functional relevance of the Jra-predicted downstream targets identified by SCENIC analysis, we focused on the Arp2/3 complex given its evolutionary conserved role in cytoskeletal remodeling across species [34,35]. Notably, Arpc1, Arp2 and Arp3 are key components of this complex, and were all predicted Jra targets, each with a high normalized enrichment score (NES = 6.33; S6C Fig). To further corroborate Jra regulation of these genes, we examined publicly available Jra ChIP-seq data from wandering-stage *Drosophila* larvae [36]. This analysis revealed a strong Jra prominent peak at the promoter of *Arp3* (S7C, S7D Fig), as well as intragenic binding sites within both *Arp2* and *Arp3* (S7B-D Fig). In contrast, *Arpc1* did not exhibit any significant Jra binding within its promoter or gene body (S7A, S7D Fig), suggesting a more direct transcriptional regulation of *Arp2* and *Arp3* by Jra. Together, these findings highlight the Arp2/3 complex as a biologically relevant candidate for investigating how Jra may control cytoskeletal dynamics during epithelial-to-NPP differentiation.

**Fig 7:**
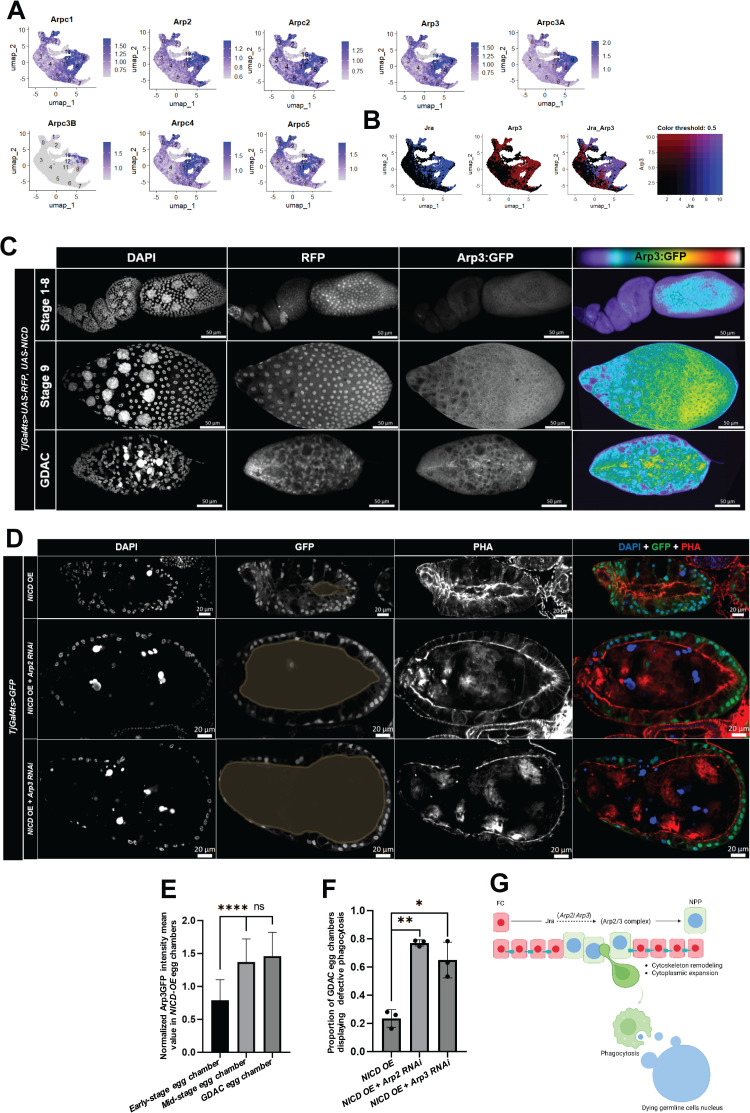
JNK regulates cytoskeleton dynamics through the Arp2/3 complex. (A) Feature Plot illustrating the gene expression of *Arpc1*, *Arp2*, *Arpc2, Arp3*, *Arpc3A*, *Arpc3B*, *Arpc4* and *Arpc5* across cell clusters in the integrated dataset. (B) Colocalization analysis showing cells co-expressing *Jra* (blue) and *Arp3* (red), with co-expressed cells highlighted in purple, in the integrated dataset. (C) *Arp3:GFP* expression in egg chambers undergoing GDAC versus control egg chambers. DAPI stains cells nuclei, PHA stains actin filaments and RFP marks the expression of *TjGal4*^*ts*^. Rainbow2 LUT (Lookup Table) was used to visualize staining intensity variations in images by mapping different pixel values to a color scale. (D) Representative confocal images of egg chambers with transgene expression in NPPs (green) expressing *NICD-*OE (Control), *Arp2RNAi* together with *NICD*-OE and *Arp3RNAi* combined with *NICD*-OE. Unengulfed germline regions are outlined with yellow dashed lines across genotypes to highlight areas not occupied by NPP cytoplasmic projections. Cells nuclei are displayed by DAPI (blue). F-actin is marked by Phalloidin (red) and *TjGal4ts* activity is shown by GFP. (E) Bar plot with error bars showing normalized *Arp3:GFP* Intensity Mean Value in *NICD*-OE egg chambers in different oogenesis stages, early stage egg chambers (stages 1-7), mid-stage egg chambers (stages 8-9), GDAC egg chambers. Sample sizes for early-stage egg chambers is N = 63, mid-stage egg chambers, N = 63, GDAC egg chambers, N = 62. P-values obtained from Mann Whitney test are indicated by **** (p ≤ 0.0001) and ns (non-significant). (F) Bar plots with error bars displaying percentage of egg chambers exhibiting defective phagocytosis in each replicate (Rep.) across different genotypes: *NICD* OE (Rep. 1, N = 54, Rep. 2, N = 43, Rep. 3, N = 57), *NICD* OE + *Arp2 RNAi* (Rep. 1, N = 89, Rep. 2, N = 102, Rep. 3, N = 173), *NICD* OE + *Arp3 RNAi* (Rep. 1, N = 141, Rep. 2, N = 73, Rep. 3, N = 60). A p-value obtained from t-test is indicated by * (p = 0.0157) and ** (p = 0.002) above bar plot. (G) Proposed working model generated in BioRender illustrating the transition of a follicle cell (FC) into a Non-Professional Phagocyte (NPP). The model highlights the regulatory role of Jra and the Arp2/3 complex, with *Arp2 and Arp3* genes encoding components of the Arp2/3 complex, predicted by SCENIC and Chip-seq to be a downstream target of Jra.

We continued our analysis by examining the expression patterns of the eight genes listed in FlyBase (flybase.org) as encoding components of the Arp2/3 complex (Fig 7A, B). Most of these genes exhibited enrichment in polar cells (cluster 1) and *NICD*-OE-derived NPPs (clusters 8 and 9). Additionally, their expression was also elevated in stretched cells (cluster 11) and in centripetal and border cells (cluster 12), specialized epithelial cell types known to undergo extensive cytoskeletal remodeling during egg chamber morphogenesis [37,38,39,40] and, in the case of centripetal and border cells, active migration [41,42,40,43] (Supplementary table 10). We also observed that the majority of *NICD*-OE-derived NPPs co-expressed *Arp3* and *Jra* in clusters 8 and 9 (Fig 7B), supporting the regulatory relationship predicted by SCENIC analysis.

To validate Arp2/3 expression in NPPs, we performed immunofluorescence using *Arp3-GFP* and confirmed its expression in both *NICD*-OE- and starvation-derived NPPs. Our results showed that *Arp3-GFP* expression gradually increases throughout oogenesis in follicle cells, reaching its peak in stage 9 egg chamber follicle cells and NPPs under both *NICD*-OE (Fig 7C, E; early-stage egg chambers, median = 0.82, N = 63; mid-stage egg chambers, median = 1.3, N = 63; and GDAC egg chambers, median = 1.4, N = 62) and starvation conditions (S8A, S8B Fig; early-stage egg chambers, median = 0.22, N = 66; mid-stage egg chambers, median = 0.44, N = 30; and GDAC egg chambers, median = 0.51, N = 24). This pattern, to some extent, resembles the gradual increase in *Jra:GFP* expression observed in egg chambers as they progress to more advanced developmental stages, ultimately culminating in NPP differentiation (S4A-D Fig).

To test the functional role of the Arp2/3 complex in NPPs, we knocked down *Arp2* and *Arp3* in *NICD*-OE-derived NPPs and starvation-induced NPPs. In both models, cytoplasmic expansion was impaired and reduced the ability of NPPs to invade the germline cell area. These defects limited the efficient removal of condensed DNA debris left by dying germline cells (Fig 7D, F; mean *NICD*-OE = 0.2343 (Replicate 1: 0.2593, N = 54, Replicate 2: 0.1628, N = 43, Replicate 3: 0.2807, N = 57), mean *NICD*-OE* + Arp2 RNAi *= 0.7708 (Replicate 1: 0.7865, N = 89, Replicate 2: 0.7450, N = 102, Replicate 3: 0.7808, N = 73), mean *NICD*-OE* + Arp3RNAi *= 0.6485, (Replicate 1: 0.5319, N = 141, Replicate 2: 0.6301, N = 73, Replicate 3: 0.7833, N = 60); S8C, S8D Fig; mean *Control* = 0.1547 (Replicate 1: 0.1346, N = 52, Replicate 2: 0.1556, N = 45, Replicate 3: 0.1739, N = 46), mean *Arp2 RNAi *= 0.641 (Replicate 1: 0.6585, N = 41, Replicate 2: 0.7435, N = 39, Replicate 3: 0.5208, N = 48), mean *Arp3 RNAi *= 0.8873 (Replicate 1: 0.8667, N = 60, Replicate 2: 0.8596, N = 57, Replicate 3: 0.9355, N = 31).

## Discussion

### Single-cell transcriptomic atlas of *NICD*-OE-induced NPPs

The ability of epithelial cells to acquire phagocytic capabilities is fundamental for maintaining tissue homeostasis by promoting the clearance of apoptotic debris. While professional phagocytes such as macrophages specialize in this function, NPPs acquire phagocytic competence in response to developmental and environmental stimuli [15,44,45]. However, the transcriptional mechanisms regulating NPP differentiation need to be better understood.

Building on our previous study [16], which demonstrated that Notch-induced polyploidization transforms follicle cells into NPPs by permitting JNK pathway activation, we expanded this work by constructing a single-cell transcriptomic atlas. To accomplish this, we analyzed two previously generated scRNA-seq datasets from our laboratory [2,1] to investigate the transcriptional changes induced by *NICD*-OE during NPP differentiation, in comparison with *wild-type* ovarian follicle cells. Unlike traditional bulk RNA-seq, scRNA-seq enables cell-type-specific analysis, allowing us to identify subpopulations, differentiation trajectories, and stage-specific gene expression dynamics within NPPs on different stages of development.

While the starvation-induced model of NPP differentiation has been widely used and previously characterized by bulk RNA-seq [46], we observed that our approach utilizing *NICD*-OE [16] offers a more robust and uniform model of NPP induction. This strategy generates a larger and more homogeneous population of NPPs (S9 Fig), facilitating high-resolution single-cell transcriptomic analysis and improving the reproducibility of NPP differentiation profiling.

We recognize, however, that *NICD* OE represents an artificial induction system. To evaluate its physiological relevance, we compared *NICD-*OE-derived NPPs with starvation-induced NPPs and found overlap in key features. Both models showed enrichment of phagocytosis-associated genes such as *crq*, *drpr*, *prtp*, and *Rac1* (Fig 2B, S3 Table), activation of autophagy (Fig 4A-F), and dependence on JNK signaling (Fig 5C-E). In addition, our analysis uncovered a novel JNK–Arp2/3 axis driving cytoskeletal remodeling (S6A-C, S7A-D Fig), which was functionally validated in both starvation- and *NICD*-OE-derived NPPs (Figs 7C-F, S8A-D). Thus, although artificial, *NICD*-OE recapitulates the essential transcriptional, signaling, and functional hallmarks of endogenous NPP differentiation.

### Identification of the transcriptional programs coordinating epithelial-to-NPP transition

Our analysis identified a heterogeneous population of cells in the *NICD*-OE dataset that transcriptomically diverged from *wild-type* follicle cells, clustering near mid-stage egg chamber-derived follicle cells while exhibiting distinct transcriptional profiles. Differential gene expression analysis revealed an enrichment of NPP-associated markers, which we validated using fluorescent reporter lines and immunofluorescence, confirming the identity of *NICD*-OE-derived NPPs.

To determine whether these cells followed a differentiation trajectory, we sub-clustered *NICD*-OE-derived NPPs and identified three transcriptomically-distinct stages. RNA velocity and pseudotime analyses indicated that NPPs undergo a stepwise differentiation process. While these approaches support a linear model of progression, the inherent heterogeneity of follicle cells suggests that alternative trajectories, including branching or parallel routes, may also contribute to NPP maturation. To further characterize the transcriptional dynamics associated with each differentiation stage, we performed differential gene expression analysis followed by Gene Ontology enrichment analysis, which identified stage-specific transcriptomic profiles defining key competences acquired at each stage of NPP differentiation.

At the earliest stage, cluster 0, the majority of expressed genes was enriched for oogenesis and follicle cell differentiation, as well as genes associated with mitochondrial energy metabolism. This transcriptomical profile suggests that these cells retain characteristics of epithelial precursors while simultaneously undergoing a metabolic shift, likely to meet the energetic demands associated with the acquisition of phagocytic capabilities by NPPs. As differentiation progressed towards cluster 1, gene expression shifted toward cytoskeletal dynamics and cell motility, matching with the stage where NPPs undergo cytoplasmic expansion toward apoptotic germline debris. Finally, in the most advanced NPP population, cluster 2, we observed an upregulation of metabolic adaptation and autophagy-related pathways, suggesting that fully differentiated NPPs reprogram their metabolism to facilitate apoptotic debris degradation.

Through the integration of regulatory network analysis with ChIP-seq data, we propose a mechanistic link in which JNK, through its downstream transcription factor Jra, regulates the expression of *Arp2* and *Arp3*, core components of the Arp2/3 complex, thereby orchestrating the cytoskeletal changes required for NPP phagocytic function. Together, these findings extend our understanding from the upstream prerequisites of NPP induction to the downstream transcriptional and cytoskeletal programs that drive full NPP differentiation.

Furthermore, resolving NPPs into distinct transcriptional states, our approach represents a major technical advancement, enabling the identification of key molecular signatures defining each phase of epithelial-to-NPP transitions. This refined model can now serve as a framework for studying NPPs in other biological contexts.

### Single-Cell approaches as a powerful tool to study NPP differentiation across model systems

The strategy of leveraging scRNA-seq analytical tools to analyze the differentiation steps of epithelial cells into NPPs could enhance our understanding on several diseases development and identify potential therapeutical targets. For example, retinal pigment epithelium (RPE) cells are originally derived from epithelial cells and have acquired specialized phagocytic capabilities to perform the phagocytosis and turnover of photoreceptor outer segments. This process occurs in three stages: binding, endocytosis, and elimination. All essential to assure that RPE cells eliminate exfoliated POS properly and maintain normal renewal of visual cells [47,48,49,50].

While scRNA-seq has been largely deployed to investigate RPE-cells [51], we believe that sub-clustering RPE-phagocytic cells and tracking shifts across differentiation stages, can be a new approach to address the RPE-transcriptomic changes based on their main phagocytic competences (binding, endocytosis and elimination). Integrating techniques such as SCENIC regulatory network analysis could help identify the transcriptional programs undergoing each stage of RPE-mediated phagocytosis, providing insights into key-gene regulators governing RPE differentiation and function.

Understanding the transcriptional mechanisms that enable RPE cells to acquire phagocytic function could provide critical insights into the molecular changes underlying defective RPE phagocytosis, which contribute to diseases such as age-related macular degeneration (AMD) [52,53], retinitis pigmentosa (RP) [52], and Stargardt disease [54].

### JNK signaling as a key regulator of NPP differentiation

JNK signaling is a conserved sub-branch of the MAPK signaling kinase present across metazoans [55,56,57,58,59]. The role of MAPK signaling kinase has been largely reported in mammalian professional and non-professional phagocytes [60,61,62,63]. Our study proposed to answer how JNK signaling orchestrate mid-stage ovarian NPP differentiation. Through SCENIC analysis, we identified, the JNK downstream transcription factor, Jra as one of the most active network associated with cell migration and cytoskeleton remodeling, required functionalities by early maturating NPPs. We confirmed that depletion of Jra resulted in impaired NPP invasion and cytoplasmic expansion, preventing cells from invade germline cell area and clearing dead-germline cells debris. On the other hand, overexpressing Jra induced follicle cells to acquire invasive and stretched morphology, supporting the role of JNK activation on driving cytoskeleton remodeling in NPPs.

Further supporting the role of JNK signaling in cytoskeletal remodeling, SCENIC, in association with ChIP-seq analysis, identified *Arp2* and *Arp3*, which encode components of the Arp2/3 complex, as part of the Jra regulon. Given that Arp2/3 is a well-known regulator of actin polymerization [64], we tested the functionality of the Arp2/3 complex on NPP differentiation by depleting two of its main components, Arp2 and Arp3, which partially impaired NPPs of performing proper cytoplasm expansion and engulfment of apoptotic debris. Consistent with these results, both MAPK signaling and the Arp2/3 complex have been implicated in coordinate cell motility and cytoskeleton rearrangements in conserved-different cell types including professional phagocytes such as neutrophils and NPPs like humans fibroblasts [65,66,67,68,69,70].

Thus our approach not only identified conserved regulators of cytoskeleton dynamics but also potentially uncovered a previously unreported transcriptional link between JNK signaling and the Arp2/3 complex that underlies actin remodeling and phagocytic competence in NPPs. This approach offers valuable insight into the mechanisms of non-professional phagocytosis and can help uncover conserved regulatory connections across diverse biological systems.

## Methods

### Fly Stocks and Husbandry

Flies were maintained under standard laboratory conditions at 25 °C and fed with yeast for three days prior to dissection. For starvation experiments, flies were fed with yeast for two days and starved for one day prior to dissection. To induce mosaic clones, we used the FLP-Out Gal4 system, with flies heat-shocked at 37 °C for 15 minutes to achieve a partial mosaic induction.

To confirm JNK pathway activation during *dJUN^CA^* overexpression in follicle cells, we used TRE-GFP as a reporter. For these experiments, *hsflp;;act>>RFP* was preferred over *TjGal4^ts^*, as it carries an non-occupied second chromosome, facilitating the insertion of TRE-GFP. In this context, flies were heat-shocked at 37 °C for 40 minutes to achieve a more dominant mosaic expression of *dJUN^CA^*. However, for quantitative evaluation of *dJUN^CA^’s* effect on follicle cell invasion of germline cells, *TjGal4^ts^* was preferred due to its more predominant expression in follicle cells compared to the *hsflp;;act>>RFP* induced mosaic system.

For temperature sensitive experiments utilizing *tub-Gal80*^*ts*^ associated with *tj-Gal4* flies crosses were expanded at 18°C, upon F1 flies’ selection, flies were fed with yeast, transferred to a 29°C incubator, and kept for 3 days for transgene induction. Following lines used in this study, *UASp-mCherry-Atg8a (#37750), UAS-Atg3-RNAi (#34359), UAS-Jra-RNAi (#31595), UAS-kayDN (#7214), Jra:GFP (#50755), kay:GFP (#38657), UAS-Arp3:GFP (#39722), UAS-Arp2-RNAi (#27705), UAS-Arp3-RNAi (#53972), UAS-GFP (*#4775), UAS-RFP (#30556), *tubP-GAL80[ts](#7019),* were ordered from Bloomington *Drosophila* Stock Center at Indiana University. *TjGal4* driver (Kyoto Stock Center, #104055).

We thank Dr. Singh for providing *UAS-dJUN^CA^* [71], Dr. Bohmann for TRE-GFP [72] and *puc-lacZ*[E69] [73], and Dr. Schüpbach for generously sharing *UAS-NICD* [74], which was designed on the *Drosophila* second chromosome. Additionally, *UAS-NICD* [75], designed on the *Drosophila* third chromosome, was generated in our lab and *hsflp;;act>>RFP* is a tool line routinely used in our lab.

### Immunofluorescence staining and imaging

Flies were dissected at room temperature in 1x PBS, followed by fixation utilizing 4% paraformaldehyde (PFA) for 15 minutes. Upon completed fixation, ovaries were washed for 30 minutes in 1x PBT (PBS with 0.2% Triton X-100). Followed by overnight incubation with primary antibody diluted in PBT. Primary-antibody stained ovaries were then washed with 1x PBT for 30 minutes, subsequently secondary antibody, diluted on PBT, was added for either overnight incubation at 4°C or 2 hours at room temperature. Secondary-antibody-stained ovaries were washed for 30 minutes with 1x PBT, followed by 10–15 minutes of DAPI staining (Invitrogen, 1 µg/mL) to stain cells nuclei. Finally, 80% glycerol mounting solution were added and samples were mounted on microscopy slides compatible with confocal miscroscopy.

For LysoTracker staining, samples were dissected in PBS. Following dissection, LysoTracker was added at a 1:1000 dilution. Samples were incubated and covered from the light for 10 minutes. After incubation, PBS was removed, and a PBS wash was performed to reduce background staining. Fix solution was then added, and staining proceeded as normally. The primary antibodies used in this study were obtained from Abcam and the Developmental Studies Hybridoma Bank (DSHB). From Abcam, we used mouse anti-ATP5A (ab14748, 1:500 dilution), Antibodies from DSHB included: mouse anti-arm (N27A1, 1:40), mouse anti-Draper (5D14,1:50), mouse anti-Fas3 (7G10,1:10), Mouse anti-β-gal (40-1a-s,1:5 dilution) and mouse anti-Dlg (4F3,1:50). The secondary antibodies were: Alexa Fluor 488, 546, and 633 (1:400, Invitrogen). Following dies were obtained from Invitrogen: Phalloidin Flour 633 (A22284), LysoTracker Red DND-99 (L7528).

### Statistics and reproducibility

Representative images, shown in the Figs, of egg chambers and their respective cells across different genotypes were acquired using Zeiss LSM 800 confocal microscopy. These images are representative of at least three independent experiments, with a minimum of 20 ovaries dissected per genetic cross. The genotypes used for each experiment are indicated in the corresponding Fig legends.

Images utilized for quantitative fluorescence analysis, intensity mean values for antibody staining, dye staining, and fluorescent proteins (e.g., GFP) were obtained using Zeiss LSM 980 confocal microscopy. To ensure reproducibility and comparability, all samples were prepared under standardized experimental conditions, and fixed imaging settings were applied across all samples. Image saturation was avoided during image taking, to ensure consistent and accurate signal acquisition.

Image analysis and processing were conducted using ZEN Blue (Zeiss). The regions of interest (ROIs) corresponding to egg chambers undergoing GDAC were manually outlined using the spline contour tool and compared to control (non-GDAC) egg chambers. Mean fluorescence intensity values for each selected ROI were recorded and analyzed using GraphPad Prism version 9.5.1 for Windows (GraphPad Software, Boston, Massachusetts, USA, www.graphpad.com). The colocalization of RFP-expressing cells with TPA response element-GFP (TRE:GFP) expression was analyzed using the colocalization toll provided by ZEN blue and the Colocalization Coefficient was selected as the method to quantify colocalization.

To ensure statistical robustness, colocalization and intensity mean values identified as outliers were excluded prior to analysis. Normality tests were then performed to determine the appropriate statistical test. If data followed a normal distribution, unpaired t-tests with Welch’s correction were applied. For data sets that did not meet the assumptions of normality, the non-parametric Mann-Whitney U test was used. In both cases, a p-value < 0.05 was considered statistically significant.

Fluorescence intensity values were normalized to DAPI mean intensity after confirming that DAPI levels did not significantly vary across samples. Bar plots representing normalized fluorescence intensity or colocalization values across genotypes were generated using GraphPad Prism. Error bars represent interquartile range (IQR) for Mann-Whitney U tests and standard error for t-tests, with corresponding p-values indicated.

### Single-cell RNA-sequencing data processing

The scRNA-seq dataset used for the analysis of *TjGal4*^*ts*^>*NICD-OE* cells was originally generated and published by our lab [1] and is publicly available in the SRA database (SRX11836918). After running Cell Ranger processing pipeline, we obtained an estimated number of 33,703 cells with an average of 10,683 reads per cell and a median of 1,014 genes per cell, with a total of 360,058,745 reads.

Similarly, the scRNA-seq dataset used for the analysis of *w^1118^* ovarian cells in this study was also originally published by our lab [2] and is publicly available in the SRA database (SRX7814226). Following Cell Ranger processing, we obtained estimated 37,928 cells with an average of 11,333 reads per cell and a median of 907 genes per cell, with a total of 459,855,892 reads.

### Cell ranger processing

Raw FASTQ files obtained from NCBI SRA (experiment accession numbers SRX11836918 and SRX7814226) were processed using Cell Ranger v7.1.0. The reference genome used for alignment was the Berkeley *Drosophila* Genome Project 6 (BDGP6.46.dna.toplevel). The Cell Ranger count pipeline was used for read alignment, filtering, barcode assignment, and unique molecular identifier (UMI) counting, generating a multidimensional feature-barcode matrix for both *w^1118^* and *NICD-OE ovarian cells*.

Following Cell Ranger processing, BAM files from the *NICD*-OE ovarian dataset were further analyzed using Velocyto for annotation of unspliced and spliced reads, following the 10x Genomics Trajectory Analysis pipeline (https://www.10xgenomics.com/analysis-guides/trajectory-analysis-using-10x-Genomics-single-cell-gene-expression-data) as described by La Manno et al. [76].

### Seurat analysis

Single-cell RNA sequencing data were processed and analyzed using Seurat v5.1.0 [17]. Quality control filtering was applied to remove low-quality cells and potential doublets/multiplets, with dataset-specific thresholds established based on gene count, UMI count, and mitochondrial gene expression.

For the *w^1118^* dataset, cells with more than 3,200 detected genes and total RNA counts exceeding 30,000 were removed to prevent the inclusion of potential doublets or multiplets. Additionally, cells exhibiting mitochondrial gene expression above 5% were excluded to filter out low-quality or dying cells.

For the *NICD*-OE dataset, an upper threshold of 3,400 genes per cell and 31,000 RNA counts per cell was applied to eliminate potential doublets or multiplets. Similar to the *w^1118^* dataset, cells with mitochondrial gene expression exceeding 5% were removed. Mitochondrial genes were identified based on the ‘mt:’ prefix in the annotation.

After preprocessing, 8,871 cells were retained for the *w^1118^* dataset, while 25,122 cells were obtained for the *Tjts>NICD*-OE dataset. The gene expression matrices for these cells were normalized, scaled, and subjected to linear dimensionality reduction following the Seurat pipeline. Cell-cycle scoring was performed using the list of cell-cycle genes proposed by [2], and cell cycle scores were regressed out during data scaling to prevent clustering and downstream analyses from being biased by cell cycle effects.

To further ensure the retention of high-quality cells, the ScDblFinder package was used to identify and remove predicted doublets. Cells were then embedded in low-dimensional space using UMAP with parameters in according to the Seurat pipeline. Cells that were displayed outside their original clusters were considered outliers and were removed to maintain cluster integrity and improve cluster visualization. As a result of these quality control steps, a final dataset of 8,037 cells was obtained for *w^1118^*, and 21,704 cells for *Tjts>NICD* OE.

To focus on follicle cell differentiation into NPPs, non-follicle cell types were identified and removed based on the expression of marker genes defined by previous studies [18,2,1,19]. After plotting follicle cell only related clusters with UMAP, additional outliers were identified and removed. Following this final refinement, the dataset consisted of 4,264 high-quality follicle cells for *w^1118^* and 17,304 high-quality follicle cells for *Tjts>NICD* OE.

To integrate *Tjts>NICD* OE and *w^1118^* follicle cells, we used the FindIntegrationAnchors() and IntegrateData() functions [77], specifying 2000 genes for anchor finding and 50 dimensions for Canonical Correlation Analysis (CCA). We selected CCA for dataset integration as it better captured shared variance while minimizing batch effects in our dataset. While Reciprocal PCA (RPCA) is computationally more efficient, CCA provided superior integration, preserving known biological clusters. Additionally, CCA was chosen over RPCA because both the *NICD*-OE and *w^1118^* datasets display sufficient overlapping features, making it an appropriate method for aligning shared cell populations while maintaining genotype-specific differences.

After integration, UMI counts in the integrated assay were normalized, log-transformed, and scaled. Cells were clustered using 40 principal components (PCs) and a resolution parameter of 0.325 was chosen. To visualize the integrated dataset, cells were projected onto a lower-dimensional UMAP space, using 50 nearest neighbors and a minimum distance of 0.7 to approximate the local manifold structure.

To perform differential gene expression (DGE) analysis, we used the “RNA” assay from each Seurat object. For integrated datasets in which the “RNA” assay contained multiple expression layers (corresponding to control and *NICD* overexpression conditions), we applied the JoinLayers() function (Seurat v5) to merge the layers into a unified expression matrix, enabling DGE analysis using FindMarkers().

To recover missing gene count values in the RNA assay, we applied ALRA imputation [78]. The imputed matrix was stored in the “alra” assay and was used for generating gene enrichment plots. To improve visualization of cluster-specific marker enrichment, the plots were scaled between the range of 5^th^ and 95^th^ quartiles.

### Postprocessing of single-cell RNA-sequencing data

#### RNA velocity analysis.

To infer the developmental progression of *NICD*-OE-derived NPPs, we applied RNA velocity-based trajectory to understand the transcriptional dynamics based on the splicing kinetics of these cells. We generated a.cloupe file, using LoupeR, from the processed Seurat object containing *NICD-OE* isolated NPP cells (10x Genomics, https://www.10xgenomics.com/support/software/loupe-browser/latest/tutorials/introduction/lb-louper). The projections and cluster identities from *NICD-OE* isolated NPP cell were exported from Loupe Browser for downstream integration with scVelo.

Velocyto [76] was excuted in a conda v.23.5.2 enviroment, and the Cell Ranger output possorted_genome_bam.bam file was used to obtain unspliced and spliced transcripts for Velocyto analysis. This analysis resulted in a.loom file containing the splicing kinetics information for each transcript across cells, which were further applied on scVelo [79] along with the Cell Ranger output filtered_feature_bc_matrix to recover RNA velocity, latent time and inferred developmental trajectories.

scVelo analysis was performed on Jupyter Notebook v.6.4.3 and cluster-specific latent time distribution was assessed by plotting *NICD*-OE isolated NPP cells on a UMAP plot along with respective RNA-velocity vectors and pseudotime values to visualize cellular-lineage progression (https://www.10xgenomics.com/analysis-guides/trajectory-analysis-using-10x-Genomics-single-cell-gene-expression-data).

#### Regulon analysis using SCENIC.

Regulon activity was analyzed using the R package SCENIC v1.3.1 [80]. The expression matrix was extracted from the “RNA” assay of the Seurat object and log-transformed before being used as input for SCENIC analysis. Seurat metadata, including cluster identities, were also incorporated into the analysis.

To infer gene regulatory networks (GRNs), we applied GENIE3 [81] to identify transcription factor (TF)–target interactions based on co-expression patterns. The v8 dm6–5kb-upstream-full-tx-11species.mc8nr motif collection was used for RcisTarget motif enrichment analysis to refine the identified TF–target interactions, selecting high-confidence direct-binding targets and defining regulons (sets of genes regulated by a given TF). Regulon activity was then quantified at the single-cell level using AUCell, which calculates the area under the curve (AUC) for each regulon, providing a measure of its activation across individual cells.

To facilitate biological interpretation, GO enrichment analysis was conducted on genes within key regulons using the clusterProfiler package, focusing on Biological Processes (BP) to identify pathways enriched in specific regulatory networks. Additionally, hierarchical clustering heatmaps were generated to display the relative regulon activity across different cell states, highlighting key TF networks driving follicle cell differentiation into NPPs.

**Proofreading and code troubleshooting**

The authors acknowledge the use of ChatGPT by Open AI for grammar review, improving readability and occasional assistance with code troubleshooting. The authors take full responsibility for the scientific content of this study.

## Supporting information

S1 Fig (A, B) Dot plots illustrating the expression of cell type-specific markers used to assign clusters to distinct ovarian cell types in both *w^1118^* (A) and *NICD*-OE datasets (B). (C) Table presenting cell specific genetic markers and their corresponding associated cell types adapted from 32.
(TIF)

S2 Fig (A, C) Confocal images of *Tj-Gal4ts, UAS-GFP > UAS-NICD* (A) and starved egg chambers (C) showing ATP5A staining, a marker of mitochondrial activity in follicle cells and NPPs. (B, D) Bar plots of normalized ATP5A intensity: mean values for *NICD* OE (B) and median values for starvation (D). Sample sizes – *NICD-OE*: GDAC (N = 73), Non-GDAC (N = 91); Starvation: GDAC (N = 80), Non-GDAC (N = 75). P-values from Mann–Whitney and t-tests are indicated as **** (p < 0.0001).
(TIF)

S3 Fig (A) Cartoon image depicting the distinct differentiation phases acquired by NPPs upon germline cell death. (A′) Mid-stage egg chamber showing follicle cells serving as NPP precursors. (A′′) Early stage of NPP differentiation, with NPPs beginning to expand their cytoplasm toward the germline region to engulf apoptotic germline debris. (A′′′) Advanced stage of differentiation, in which NPPs have fully surrounded and engulfed the germline cells, displaying completed phagocytosis of germline cell debris.
(TIF)

S4 Fig (A) Jra:GFP expression in GDAC egg chambers compared to stage 1–8 egg chambers under *NICD* OE. DAPI stains cells nuclei, PHA stains actin filaments and RFP marks the expression of *TjGal4*^*ts*^. Rainbow2 LUT (Lookup Table) was used to visualize staining intensity variations in images by mapping different pixel values to a color scale. (B) Bar plot with error bars showing normalized Jra:GFP Intensity Mean Value in *NICD*-OE, sample sizes and group medians were as follows: early-stage egg chambers, median = 0.5322, *N* = 71; mid-stage egg chambers, median = 0.7363, *N* = 71; and GDAC egg chambers, median = 1.355, *N* = 71. (C) Comparison of Jra:GFP expression in GDAC egg chambers and stage 1–8 egg chambers under starvation. The Rainbow2 Lookup Table (LUT) was applied to improve visualization of intensity differences by mapping pixel values to a color gradient. (D) Bar plot with error bars showing normalized Jra:GFP intensity mean value in starved egg chambers, early-stage egg chambers, median = 0.7210, *N* = 44; mid-stage egg chambers, median = 0.9150, *N* = 45; and GDAC egg chambers, median = 1.515, *N* = 22. P-values obtained from Mann Whitney test are indicated by **** (p < 0.0001) above each plot.
(TIF)

S5 Fig (A) Mosaic clones expressing *Jra RNAi* generated by *hsflp; TRE:GFP; act>>RFP.* DAPI marks nuclei, RFP labels mosaic clones, and TRE:GFP indicates JNK pathway activation. (B) Colocalization coefficient of RFP-positive cells co-expressing TRE:GFP in control versus *Jra RNAi* mosaic cells from starved egg chambers undergoing germline cell death. Control egg chambers (median = 0.95, *n* = 34) and *Jra* RNAi egg chambers (median = 0.66, *n* = 35); **** indicates *p* < 0.0001. (C) Bar plot with error bars showing the percentage of ovarioles displaying follicle cells with an invasive germline cell phenotype in *Tj-GAL4*^*ts*^ controls versus *dJUN*^*CA*^-expressing cells under normal feeding conditions. No invasive phenotype was observed in *TjGal4ts* ovarioles (Replicate 1: N = 62; Replicate 2: N = 37; Replicate 3: N = 55). In contrast, *dJUN*^*CA*^-expressing ovarioles exhibited consistent invasive phenotype across replicated (Replicate 1: 0.9434, *N* = 53; Replicate 2: 0.9535, *N* = 43; Replicate 3: 0.9487, *N* = 39). Statistical significance was assessed by unpaired t-test; **** indicates p ≤ 0.0001.
(TIF)

S6 Fig (A, B) Gene Ontology (GO) network analysis of Jra regulon genes. (A) Bar plot summarizing GO enrichment analysis for Jra regulon genes. The x-axis shows the gene count, and the y-axis lists enriched GO biological processes. Bar length indicates the number of genes associated with each term, and the color gradient represents the adjusted p-value (p.adjust), with darker shades indicating higher statistical significance. (B) Category-net plot (Cnetplot) Nodes represent biological processes, color-coded by category, with node size reflecting the number of genes involved. Edges depict functional relationships between genes and processes. (C) Table listing the genes (gene) predicted to be regulated by the transcription factor Jra (TF), as identified by SCENIC. For each gene, the best-matching binding motif (bestMotif) from the JASPAR database (MA0491.1), the normalized enrichment score (NES), and the motif search window size (motifDb), covering 5 kb upstream and downstream of the transcription start site, are shown.
(TIF)

S7 Fig (A–C) UCSC Genome Browser views showing Jra ChIP-seq signal and peak calls at the genomic loci of *Arpc1* (A), *Arp2* (B), and *Arp3* (C) in *Drosophila melanogaster* (assembly dm6). The blue track represents Jra ChIP-seq signal intensity (ENCFF314JDI, bigWig format). Green and orange tracks show Jra ChIP-seq peak calls from two independent datasets (ENCFF200SQG, bigBed; and ENCFF740MEO, bed, respectively). RefSeq gene annotations (dm6) are shown in dark green at the bottom. (D) Table displaying the results of BEDTolls intersection analysis between ChIP-seq peaks (ENCFF857SSH) and the genetic loci and promoter regions (5kb upstream) of *Arpc1*, *Arp2* and *Arp3*. Chromosome location (Chr location) displays the chromosome in which genes are located. Region type classifies the region where the peak was detected regarding the gene either on the gene body or in the promoter region (5kb upstream). Region Start and Region End marks the region where the Jra peak starts and ends. Peak location states the chromosome region where the peak was located. Signal value displays the intensity of the ChIP-seq signal, “NA” indicates no peak detected. The q-value is displayed in the log-transformed format and represents the statistical confidence of each detected ChIP-seq peak. Peak Type classifies the region where the peak was located as intragenic promoter or undetected.
(TIF)

S8 Fig (A) *Arp3:GFP* expression in starved egg chambers displaying GDAC versus control conditions. DAPI stains cells nuclei, PHA stains actin filaments and *RFP* marks the expression of *TjGal4ts*. Rainbow2 LUT (Lookup Table) was used to visualize staining intensity variations in images by matching different pixel values to a color scale. (B) Bar plot with error bars showing normalized *Arp3:GFP* Intensity Mean Value in starved egg chambers in different oogenesis stages, early-stage egg chambers (stages 1–7), mid-stage egg chambers (stages 8–9), GDAC egg chambers. Sample sizes for early-stage egg chambers is N = 66, mid-stage egg chambers, N = 30, GDAC egg chambers, N = 24. P-values obtained from Mann Whitney test are indicated by **** (p ≤ 0.0001) and ns (non-significant). (C) Bar plot with error bars displaying percentage of egg chambers exhibiting defective phagocytosis in each replicate (Rep.) across different genotypes: *Control* (Rep. 1, N = 52, Rep. 2, N = 45, Rep. 3, N = 46), *Arpc2 RNAi* (Rep. 1, N = 41, Rep. 2, N = 39, Rep. 3, N = 48), *Arpc3 RNAi* (Rep. 1, N = 60, Rep. 2, N = 57, Rep. 3, N = 31). A p-value obtained from t-test is indicated by * (p = 0.0151), *** (p = 0.0002) above bar plot. (D) Representative confocal images of *Control* versus *Arpc2-RNAi* and *Arpc3-RNAi* expressing egg chambers. Yellow dashed lines across genotypes highlight unengulfed germline regions not occupied by NPP cytoplasmic projections. Cells nuclei is displayed by DAPI. F-actin is marked by Phalloidin and *TjGal4ts* activity is shown by GFP.
(TIF)

S9 Fig (A) Confocal images of ovaries subjected to starvation or *NICD*-OE treatment. Red “X” marks indicate GDAC egg chambers containing residual condensed DNA, a hallmark of germline cell death. DAPI stains nuclei, and GFP marks follicle cells expressing the *TjGal4ts* driver. (B) Bar plot with error bars showing the average number of GDAC egg chambers per ovary under *Starvation* (Mean = 1.6, Rep. 1 mean: 1.7, N = 21, Rep. 2 mean: 1.5, N = 21, Rep. 3 mean: 1.6, N = 24, Rep. 4 mean: 1.6, N = 15) versus *NICD* OE (Mean = 7.8, Rep. 1 mean: 15.1, N = 17, Rep. 2 mean: 8.1, N = 18, Rep. 3 mean: 7.5, N = 12, Rep. 4 mean: 7.0, N = 15). A p-value obtained from an unpaired t-test is indicated by *** (p = 0.0003) above the plot.
(TIF)

S1 Table Marker genes for non-epithelial cells in the *w^1118^* dataset. (CSV)

S2 Table Marker genes for non-epithelial cells in the *NICD*-OE dataset. (CSV)

S3 Table Marker genes for NPPs identified in clusters 8 and 9. (CSV)

S4 Table Gene expression profile of NPP cluster 2. (CSV)

S5 Table GO terms enriched among up-regulated genes in NPP cluster 0. (CSV)

S6 Table GO terms enriched among up-regulated genes in NPP cluster 1. (CSV)

S7 Table GO terms enriched among up-regulated genes in NPP cluster 2. (CSV)

S8 Table SCENIC regulon activity scores (raw versus scaled). (CSV)

S9 Table GO terms enriched among up-regulated genes predicted to be targeted by Jra. (CSV)

S10 Table Expression of Arp2/3 complex-related genes in NPPs compared with other clusters. (CSV)
