# Supplementary figures and images for: Single-cell transcriptomics reveals stepwise transformation of epithelial cells into Non-Professional Phagocytes
Source: PLoS Genet. 2025 Dec 4;21(12):e1011953. doi: 10.1371/journal.pgen.1011953 (PMC12677531; doi:10.1371/journal.pgen.1011953)

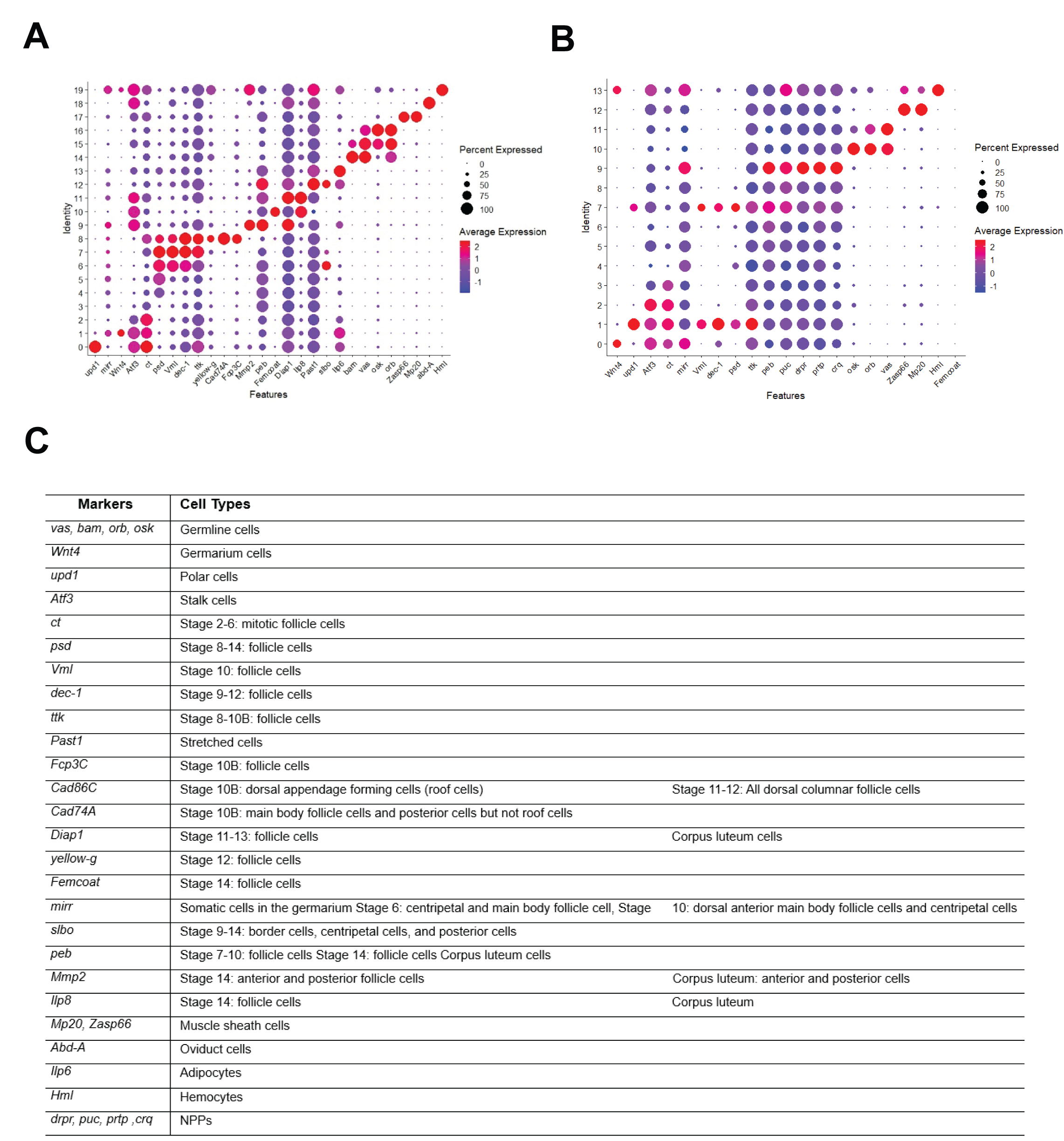

Supplement: S1 Fig — (C) Table presenting cell specific genetic markers and their corresponding associated cell types adapted from 32. (TIF) [file pgen.1011953.s001.tif]

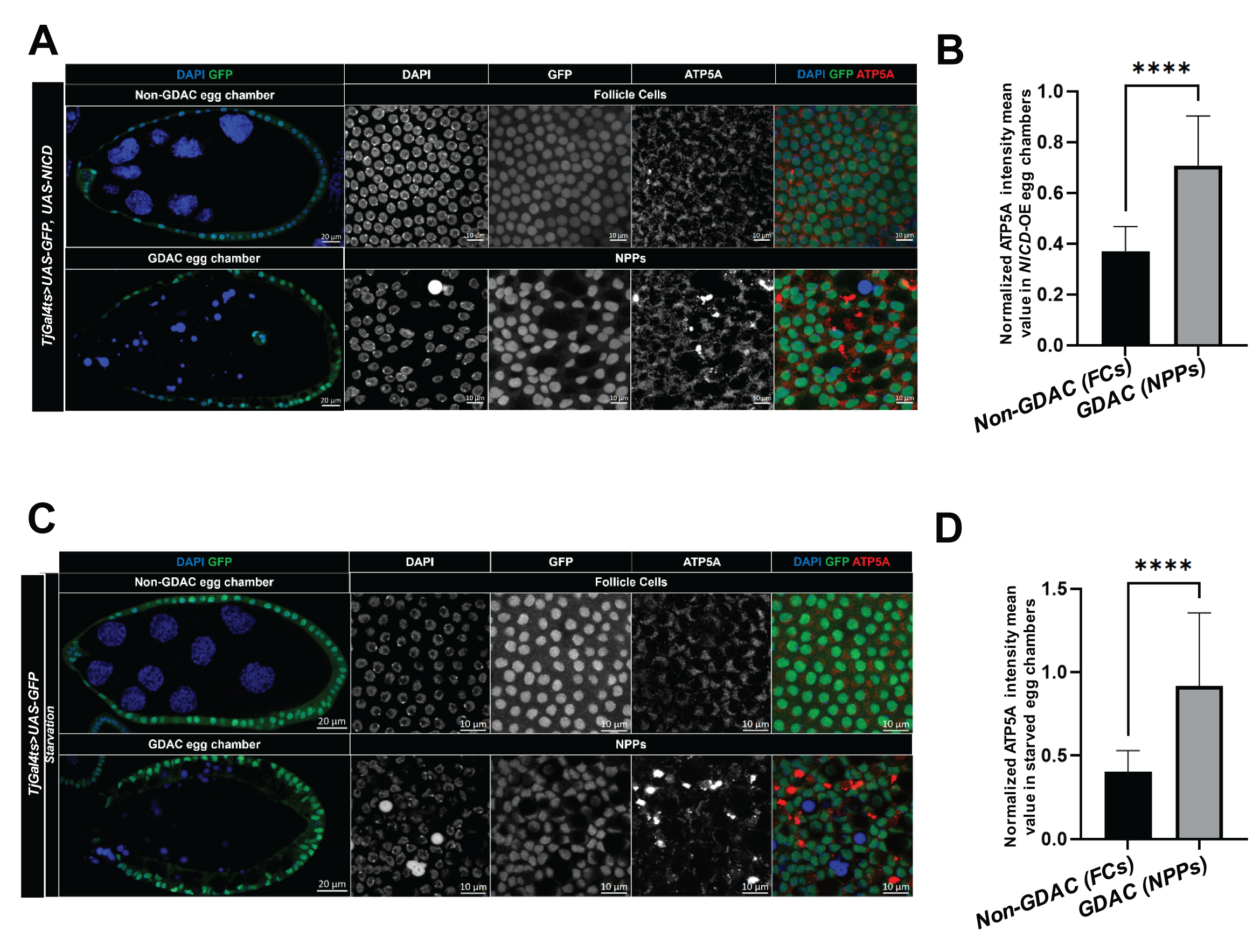

Supplement: S2 Fig — (B, D) Bar plots of normalized ATP5A intensity: mean values for NICD OE (B) and median values for starvation (D). Sample sizes – NICD-OE: GDAC (N = 73), Non-GDAC (N = 91); Starvation: GDAC (N = 80), Non-GDAC (N = 75). P-values from Mann–Whitney and t-tests are indicated as **** (p < 0.0001). (TIF) [file pgen.1011953.s002.tif]

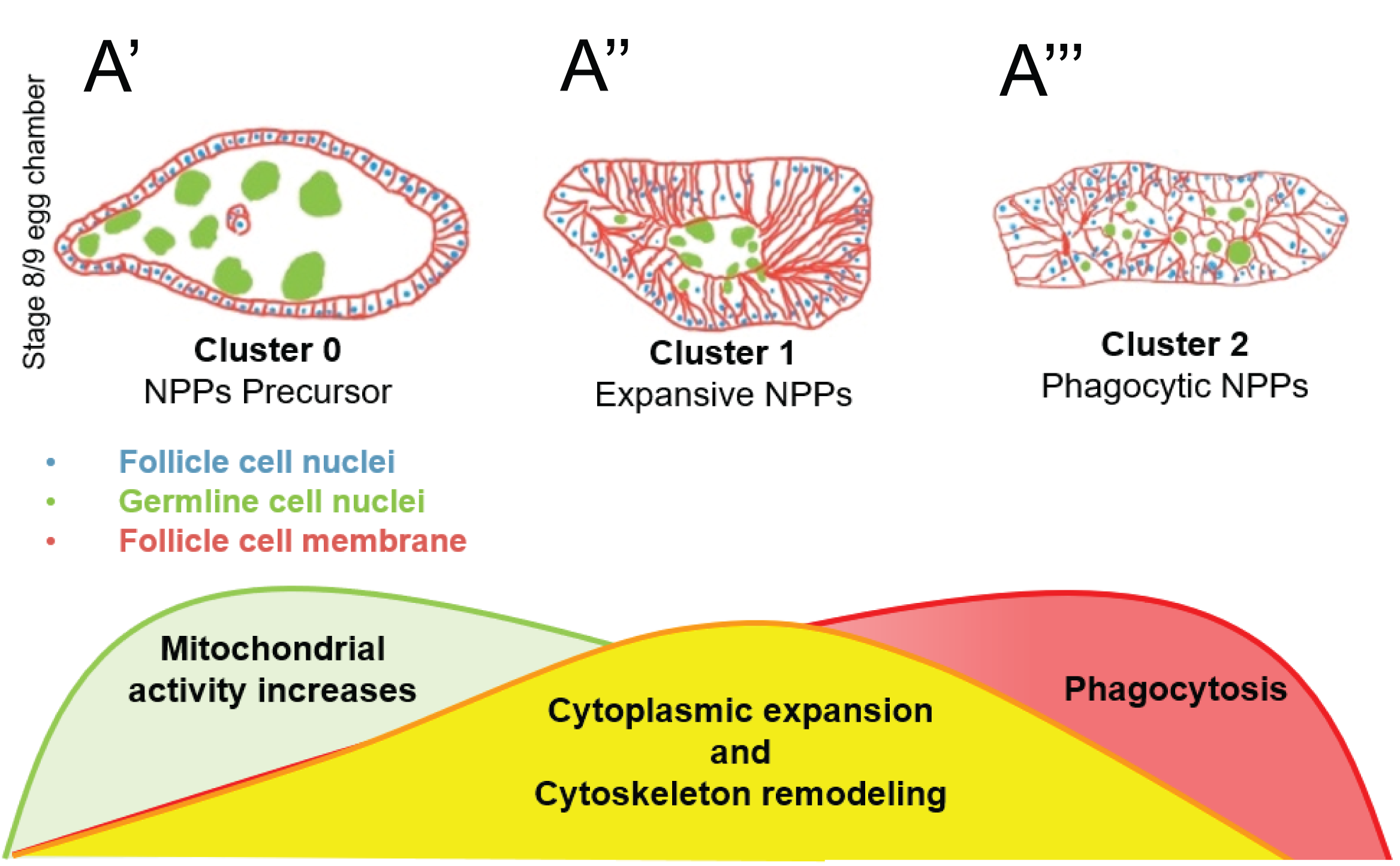

Supplement: S3 Fig — (A′) Mid-stage egg chamber showing follicle cells serving as NPP precursors. (A′′) Early stage of NPP differentiation, with NPPs beginning to expand their cytoplasm toward the germline region to engulf apoptotic germline debris. (A′′′) Advanced stage of differentiation, in which NPPs have fully surrounded and engulfed the germline cells, displaying completed phagocytosis of germline cell debris. (TIF) [file pgen.1011953.s003.tif]

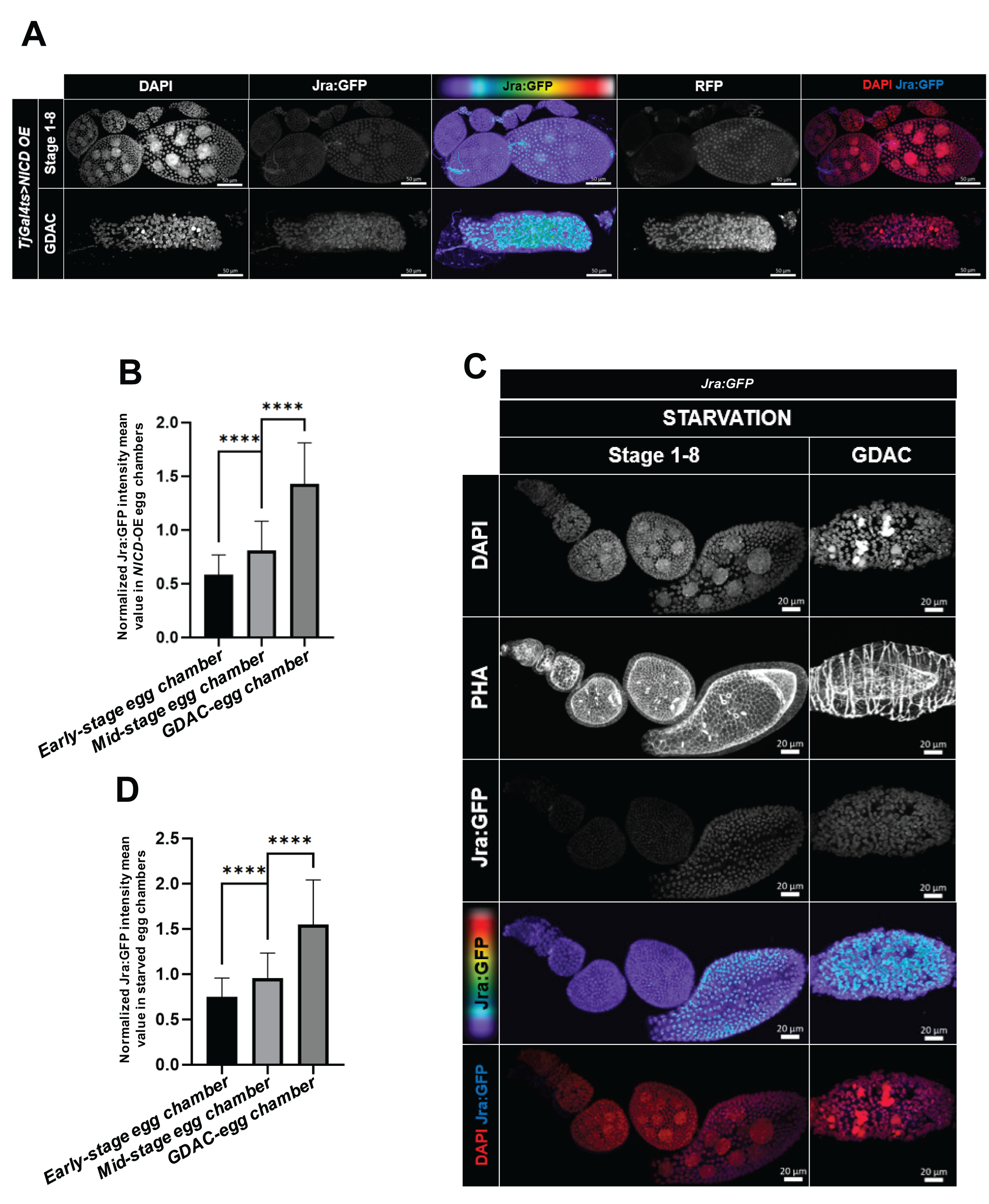

Supplement: S4 Fig — DAPI stains cells nuclei, PHA stains actin filaments and RFP marks the expression of TjGal4ts. Rainbow2 LUT (Lookup Table) was used to visualize staining intensity variations in images by mapping different pixel values to a color scale. (B) Bar plot with error bars showing normalized Jra:GFP Intensity Mean Value in NICD-OE, sample sizes and group medians were as follows: early-stage egg chambers, median = 0.5322, N = 71; mid-stage egg chambers, median = 0.7363, N = 71; and GDAC egg chambers, median = 1.355, N = 71. (C) Comparison of Jra:GFP expression in GDAC egg chambers and stage 1–8 egg chambers under starvation. The Rainbow2 Lookup Table (LUT) was applied to improve visualization of intensity differences by mapping pixel values to a color gradient. (D) Bar plot with error bars showing normalized Jra:GFP intensity mean value in starved egg chambers, early-stage egg chambers, median = 0.7210, N = 44; mid-stage egg chambers, median = 0.9150, N = 45; and GDAC egg chambers, median = 1.515, N = 22. P-values obtained from Mann Whitney test are indicated by **** (p < 0.0001) above each plot. (TIF) [file pgen.1011953.s004.tif]

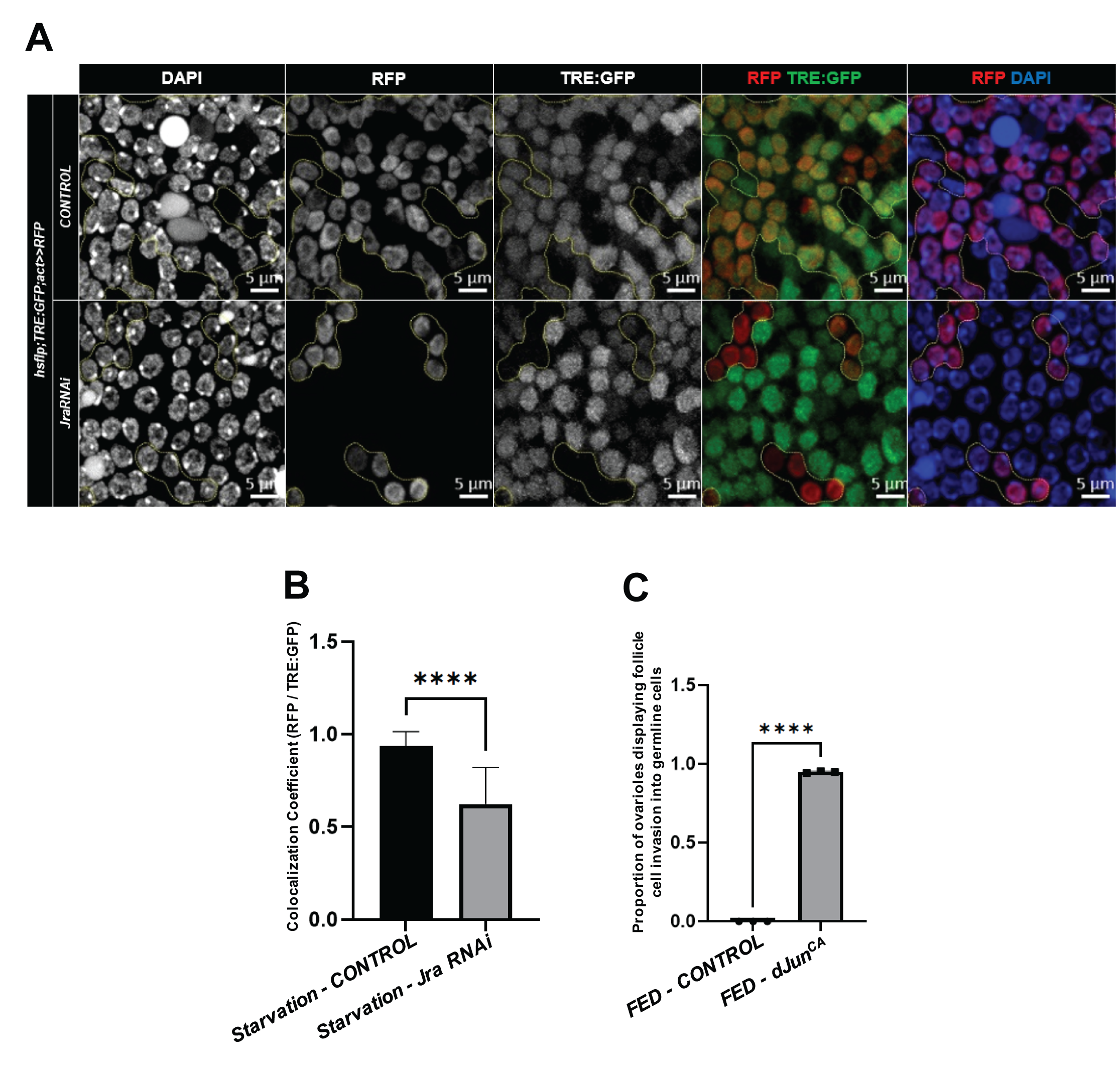

Supplement: S5 Fig — DAPI marks nuclei, RFP labels mosaic clones, and TRE:GFP indicates JNK pathway activation. (B) Colocalization coefficient of RFP-positive cells co-expressing TRE:GFP in control versus Jra RNAi mosaic cells from starved egg chambers undergoing germline cell death. Control egg chambers (median = 0.95, n = 34) and Jra RNAi egg chambers (median = 0.66, n = 35); **** indicates p < 0.0001. (C) Bar plot with error bars showing the percentage of ovarioles displaying follicle cells with an invasive germline cell phenotype in Tj-GAL4ts controls versus dJUNCA-expressing cells under normal feeding conditions. No invasive phenotype was observed in TjGal4ts ovarioles (Replicate 1: N = 62; Replicate 2: N = 37; Replicate 3: N = 55). In contrast, dJUNCA-expressing ovarioles exhibited consistent invasive phenotype across replicated (Replicate 1: 0.9434, N = 53; Replicate 2: 0.9535, N = 43; Replicate 3: 0.9487, N = 39). Statistical significance was assessed by unpaired t-test; **** indicates p ≤ 0.0001. (TIF) [file pgen.1011953.s005.tif]

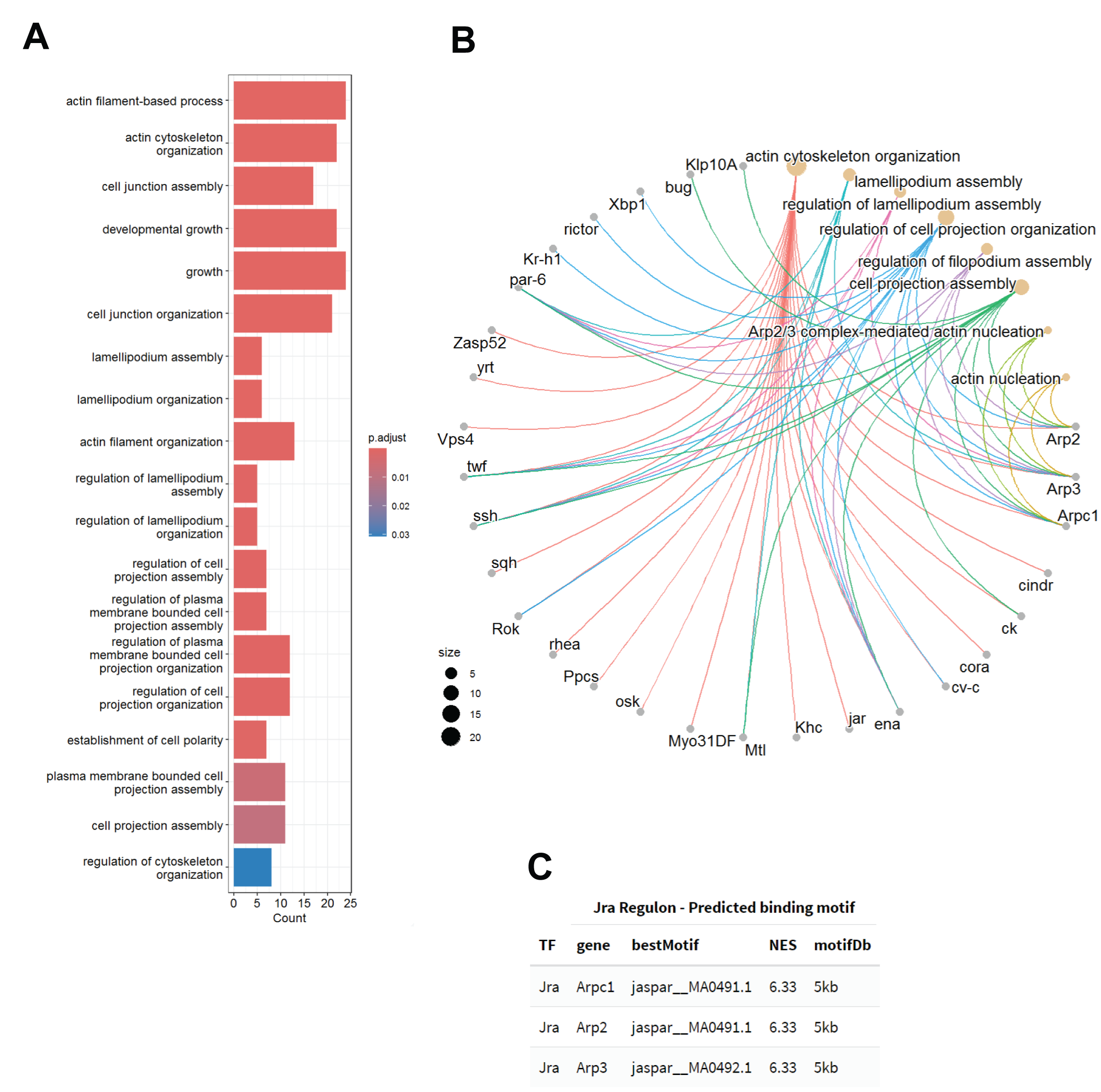

Supplement: S6 Fig — (A) Bar plot summarizing GO enrichment analysis for Jra regulon genes. The x-axis shows the gene count, and the y-axis lists enriched GO biological processes. Bar length indicates the number of genes associated with each term, and the color gradient represents the adjusted p-value (p.adjust), with darker shades indicating higher statistical significance. (B) Category-net plot (Cnetplot) Nodes represent biological processes, color-coded by category, with node size reflecting the number of genes involved. Edges depict functional relationships between genes and processes. (C) Table listing the genes (gene) predicted to be regulated by the transcription factor Jra (TF), as identified by SCENIC. For each gene, the best-matching binding motif (bestMotif) from the JASPAR database (MA0491.1), the normalized enrichment score (NES), and the motif search window size (motifDb), covering 5 kb upstream and downstream of the transcription start site, are shown. (TIF) [file pgen.1011953.s006.tif]

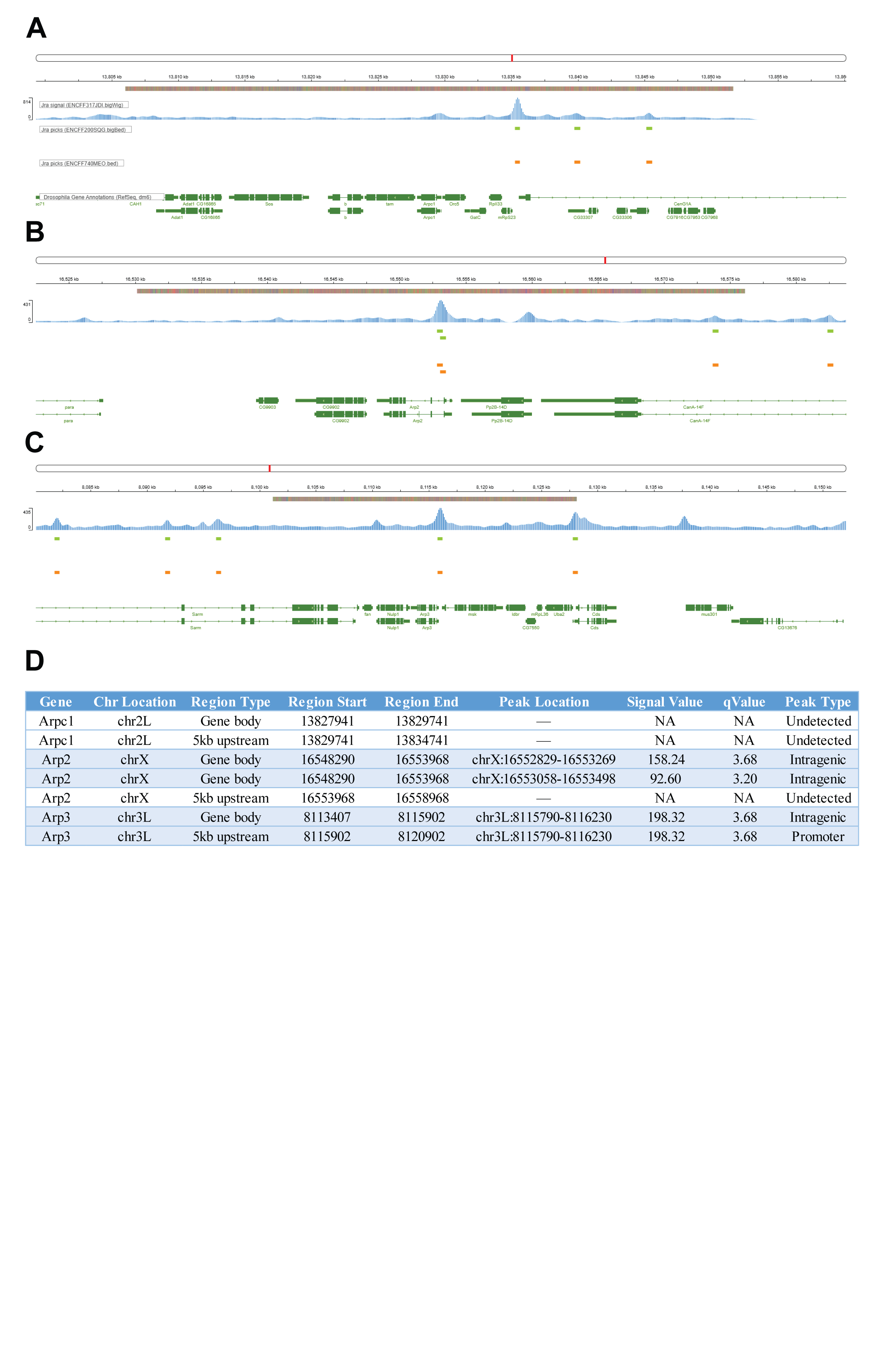

Supplement: S7 Fig — The blue track represents Jra ChIP-seq signal intensity (ENCFF314JDI, bigWig format). Green and orange tracks show Jra ChIP-seq peak calls from two independent datasets (ENCFF200SQG, bigBed; and ENCFF740MEO, bed, respectively). RefSeq gene annotations (dm6) are shown in dark green at the bottom. (D) Table displaying the results of BEDTolls intersection analysis between ChIP-seq peaks (ENCFF857SSH) and the genetic loci and promoter regions (5kb upstream) of Arpc1, Arp2 and Arp3. Chromosome location (Chr location) displays the chromosome in which genes are located. Region type classifies the region where the peak was detected regarding the gene either on the gene body or in the promoter region (5kb upstream). Region Start and Region End marks the region where the Jra peak starts and ends. Peak location states the chromosome region where the peak was located. Signal value displays the intensity of the ChIP-seq signal, “NA” indicates no peak detected. The q-value is displayed in the log-transformed format and represents the statistical confidence of each detected ChIP-seq peak. Peak Type classifies the region where the peak was located as intragenic promoter or undetected. (TIF) [file pgen.1011953.s007.tif]

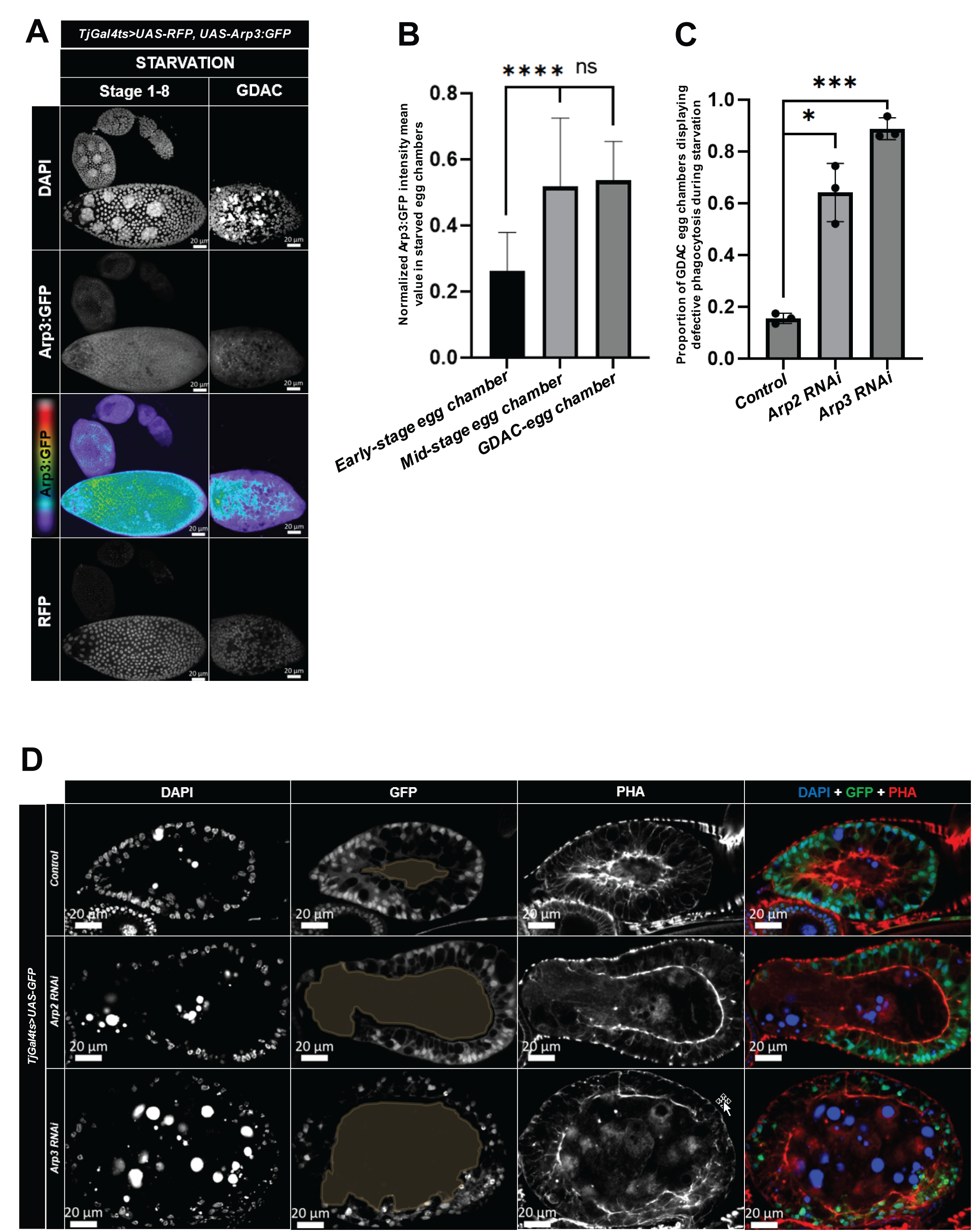

Supplement: S8 Fig — DAPI stains cells nuclei, PHA stains actin filaments and RFP marks the expression of TjGal4ts. Rainbow2 LUT (Lookup Table) was used to visualize staining intensity variations in images by matching different pixel values to a color scale. (B) Bar plot with error bars showing normalized Arp3:GFP Intensity Mean Value in starved egg chambers in different oogenesis stages, early-stage egg chambers (stages 1–7), mid-stage egg chambers (stages 8–9), GDAC egg chambers. Sample sizes for early-stage egg chambers is N = 66, mid-stage egg chambers, N = 30, GDAC egg chambers, N = 24. P-values obtained from Mann Whitney test are indicated by **** (p ≤ 0.0001) and ns (non-significant). (C) Bar plot with error bars displaying percentage of egg chambers exhibiting defective phagocytosis in each replicate (Rep.) across different genotypes: Control (Rep. 1, N = 52, Rep. 2, N = 45, Rep. 3, N = 46), Arpc2 RNAi (Rep. 1, N = 41, Rep. 2, N = 39, Rep. 3, N = 48), Arpc3 RNAi (Rep. 1, N = 60, Rep. 2, N = 57, Rep. 3, N = 31). A p-value obtained from t-test is indicated by * (p = 0.0151), *** (p = 0.0002) above bar plot. (D) Representative confocal images of Control versus Arpc2-RNAi and Arpc3-RNAi expressing egg chambers. Yellow dashed lines across genotypes highlight unengulfed germline regions not occupied by NPP cytoplasmic projections. Cells nuclei is displayed by DAPI. F-actin is marked by Phalloidin and TjGal4ts activity is shown by GFP. (TIF) [file pgen.1011953.s008.tif]

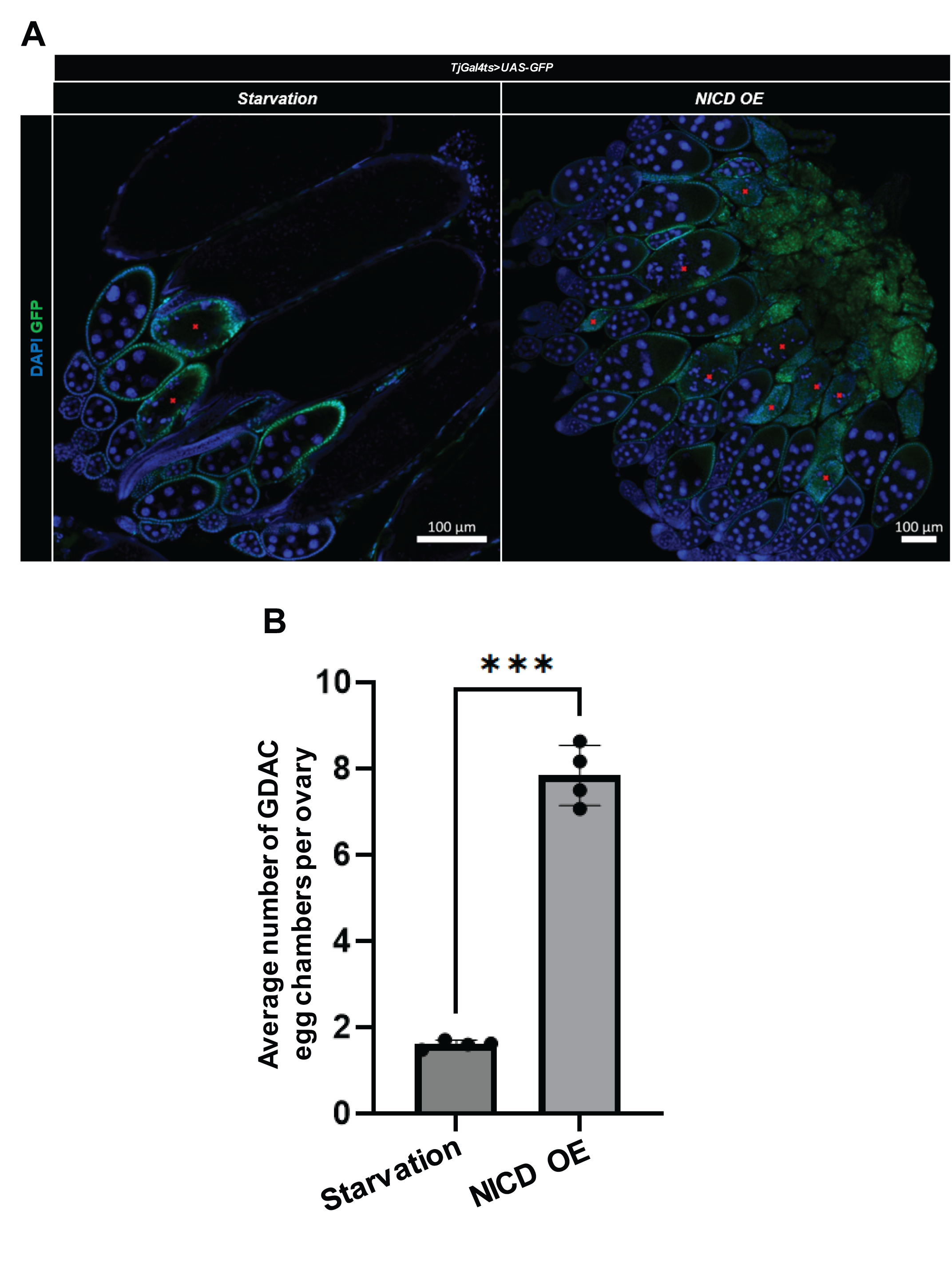

Supplement: S9 Fig — Red “X” marks indicate GDAC egg chambers containing residual condensed DNA, a hallmark of germline cell death. DAPI stains nuclei, and GFP marks follicle cells expressing the TjGal4ts driver. (B) Bar plot with error bars showing the average number of GDAC egg chambers per ovary under Starvation (Mean = 1.6, Rep. 1 mean: 1.7, N = 21, Rep. 2 mean: 1.5, N = 21, Rep. 3 mean: 1.6, N = 24, Rep. 4 mean: 1.6, N = 15) versus NICD OE (Mean = 7.8, Rep. 1 mean: 15.1, N = 17, Rep. 2 mean: 8.1, N = 18, Rep. 3 mean: 7.5, N = 12, Rep. 4 mean: 7.0, N = 15). A p-value obtained from an unpaired t-test is indicated by *** (p = 0.0003) above the plot. (TIF) [file pgen.1011953.s009.tif]
